# Strong and Agile Wall-Climbing Robots Capable of Traversing Obstacles via Anisotropic Acoustic Adhesion

**DOI:** 10.34133/research.1038

**Published:** 2026-01-07

**Authors:** Kanglong Yuan, Jun Peng, Ao Qin, Wenwu Zhu, Yikun Liu, Jiliang Ma, Yusen Ma, Xuefeng Chen, G. Jeffrey Snyder

**Affiliations:** ^1^ School of Mechanical Engineering, Xi’an Jiaotong University, Xi’an 710049, China.; ^2^ Department of Materials Science and Engineering, Northwestern University, Evanston, IL 60208, USA.

## Abstract

Small inspection robots are highly desirable for inspecting complex machinery and detecting damage in confined spaces. However, common climbing robots that rely on vacuum suction or bioinspired dry adhesion often suffer from bulky sizes or slow locomotion speeds. Developing compact yet intelligent wall-climbing robots that mimic the agility and payload capacity of geckos remains an important challenge. In this work, we design a 20-g, 10-cm artificial intelligence (AI)-integrated robot capable of carrying a 70-g payload while climbing on vertical and inverted surfaces at a speed of 70 mm/s. Acoustic adhesion is generated by vibrating a flexible annular disk on smooth surfaces, where air is periodically absorbed and expelled, resulting in negative pressure. The thin air layer with negative pressure indicates anisotropic performance, characterized by strong normal adhesion and negligible tangential resistance, making it highly suitable for designing small, yet strong, climbing robots. The theoretical model and laser surface morphology measurements reveal the thickness-dependent adhesion of a thin air layer beneath the disk. A servo-spring system is designed to meet the stringent requirements of a thin air layer thickness, yielding robust normal adhesion. Resonance analysis and the use of proper spring material stiffness further enhance adhesion performance. Therefore, combining this innovative acoustic adhesion with optimized structural design, our robot achieves gecko-like mobility and payload capacity. Additionally, integrated AI techniques simplify robot control, allowing voice-commanded operation and autonomous task execution. We demonstrate the functions of these climbing robots through agile inspections in a 3-dimensional maze and retired aircraft engines. This work presents the design of small, strong, and agile climbing robots that utilize anisotropic acoustic adhesions, demonstrating agile mobility across gaps, right corners, and discontinuous curved surfaces. It offers potential solutions for in situ damage detection in aero-engines and other complex equipment cavities.

## Introduction

Wall-climbing robots are required for equipment inspection [1–4] and maintenance [5], as well as search and rescue [6,7], particularly in confined, narrow spaces or hazardous environments. Small wall-climbing robots are compact designs to carry cameras, sensors, power sources, communication systems, and information processing devices [8–10]. They are equipped with an essential wall-climbing adhesion unit, enabling infrastructure inspection and detection of mechanical damage.

A proper adhesion mechanism is crucial for enhancing mobility and loading capacity to meet practical demands. Advanced adhesion strategies for robotic climbing locomotion on continuous flat or curved surfaces can be categorized into 4 primary mechanisms: biomimetic dry adhesion [11–13], magnetic adhesion [14–17], electrostatic adhesion [18–20], and vacuum suction [21–23]. The gecko-inspired dry adhesion enables the robot to employ van der Waals forces for wall climbing [11]. Artificial microstructures mimic the setae of geckos, typically using polydimethylsiloxane (a silicone-based organic polymer), which lacks the self-cleaning capability of geckos. The microstructures can generate adhesion on walls, but they also absorb dust from the walls, thereby resulting in loss of adhesion [18,24]. To maintain adhesion, quadrupedal and caterpillar-inspired soft robots have demonstrated exceptional adaptability to vertical and inverted surfaces through permanent magnetic attraction [16] or electrostatic adhesion [25]. While magnet-based systems exhibit remarkable payload capacities, their applications remain constrained due to their large size and the strict requirements of a ferromagnetic substrate [3,9].

Electrostatic adhesion-based robots can achieve centimeter-scale sizes and crawl on both conductive and nonconductive surfaces [19,26,27]. This adhesion mechanism requires external high voltages [28,29] to polarize nonconductive substrates, resulting in limited loading capacity, typically 1 to 5 g [19,28,30] and a slow-moving speed. In addition, small vacuum cups can achieve strong normal adhesion on both flat and curved surfaces, but the tangential friction is also strong [21,22], hindering mobility. The vacuum system remains bulky due to the necessity of an air pump and tubing. To navigate obstacles and gaps, the inchworm locomotion design has been adopted for wall-climbing robots [4,31–34]. However, slow climbing speed and limited load capacity severely restrict the mobility and applicability of crawling robots. Therefore, a novel adhesion method is urgently needed to balance robot size, substantial load capabilities, and obstacle-traversing capabilities.

Quickly lifting a flat disk on a smooth surface results in a resisting force, which is negative pressure (a partial vacuum) between the disk and the substrate surface, known as the Stefan force [35–38]. However, the negative pressure decays rapidly as the surrounding fluid (air) rushes in as the disk is separated. Obtaining continuous negative pressure requires preventing air from rushing in and continuously pumping out air during lifting. A disk vibrating at acoustic frequencies repeatedly intakes and squeezes air. When more air is squeezed out than is inhaled in a vibration cycle, an averaged negative pressure is achieved, leading to an adhesion force [38]. Conversely, this indicates repulsion or levitation [39–43]. While a vacuum suction cup exhibits robust adhesion, strong tangential friction firmly anchors it to the substrate. The vibrating disk, on the other hand, has weak, if any, lateral resistance and can slide and float like an air-hockey puck due to the compressed air layer. This acoustic adhesion thus has an anisotropic performance, characterized by strong normal adhesion and weak tangential friction, which is particularly well-suited for the design of climbing robots. Weston-Dawkes et al. [38] demonstrated a circular-shaped climbing robot based on this vibrating disk and proposed a gas-lubrication theory to explain the phenomenon. A 14-cm, 60-g robot, powered by a 1-W motor, successfully carried a 440-g payload on inverted surfaces, highlighting the promise of this novel adhesion strategy. Building on these fundamentals, optimized adhesion and, accordingly, engineering design can further improve load capacity and mobility, even enabling traversal across uneven surfaces. Therefore, anisotropic acoustic adhesion offers a potential solution for designing small climbing robots, facilitating their practical applications as inspection robots.

In this paper, we present the design of an artificial intelligence (AI)-integrated climbing robot that utilizes optimized anisotropic acoustic adhesion. The robot is capable of climbing stairs and navigating corners on vertical and inverted surfaces, demonstrating agility with a maximum speed of 70 mm/s and carrying loads exceeding 3 times its own weight. We experimentally analyzed the effects of disk dimensions and vibration frequency on the adhesion force. We revealed that the adhesion critically depends on the thickness of the air layer between the disk and substrate. A linear servo-spring system was accordingly designed to adjust the thickness of this air layer, thereby enhancing the adhesion force. The mechanical resonance of the robot and the vibrating disk were analyzed to quantify climbing risk factors. The climbing and intelligent detection functions of the wireless-controlled robot were demonstrated using a homemade 3-dimensional (3D) maze and a retired aero engine.

Our work advances understanding and engineering of anisotropic acoustic adhesion by demonstrating the design of a small yet strong and agile wall-climbing robot that can promote techniques and applications of robot-intelligent detection.

## Results

### Design principles of climbing robots

**Fig. 1.**
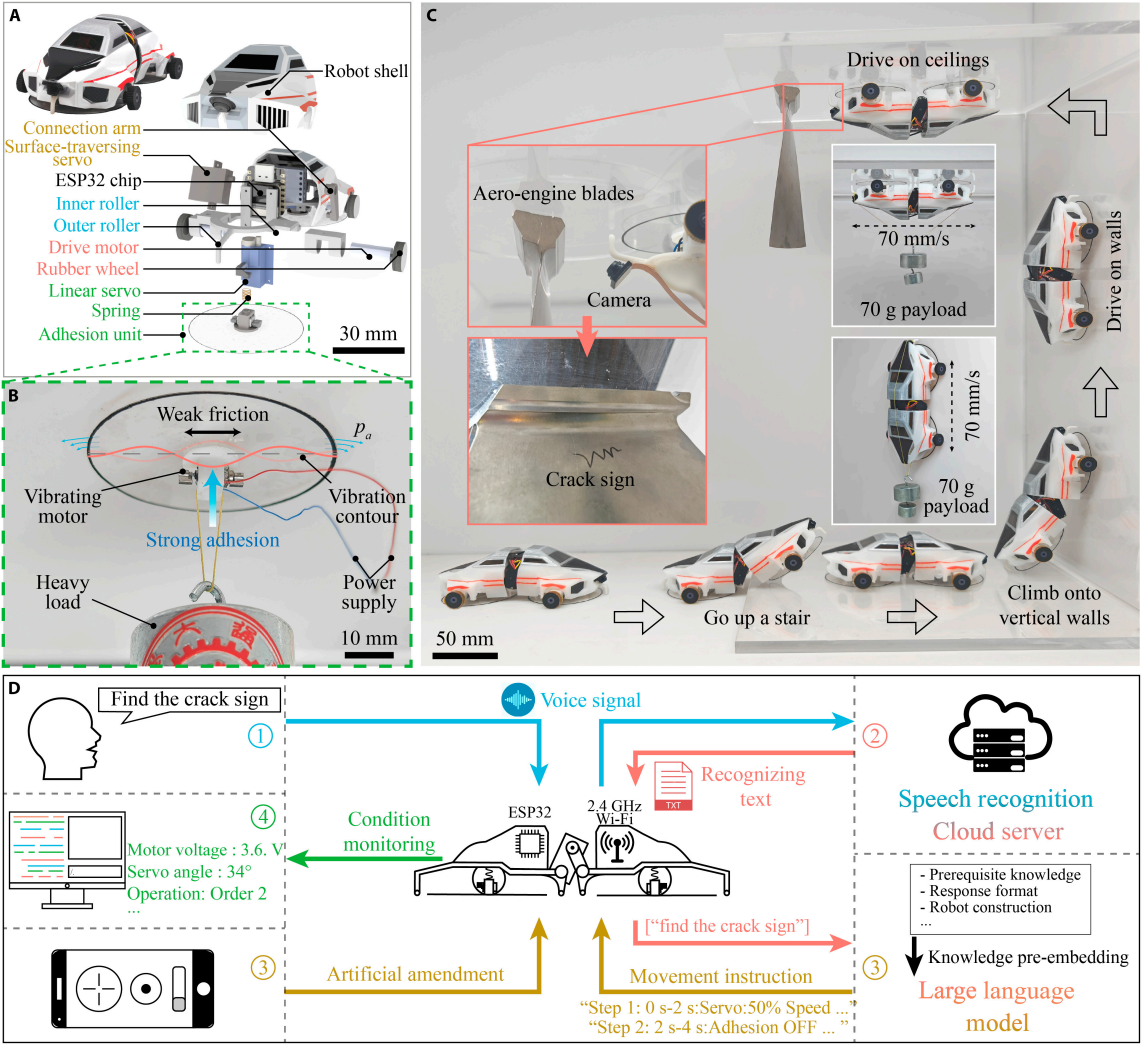
Structure and control of a climbing robot using anisotropic acoustic adhesion. (A) Photograph and 3D diagram of a 20-g robot (104 mm × 57 mm × 27 mm) with 2 vibration units (50 mm in diameter) to generate anisotropic acoustic adhesion. The robot features 4 motorized wheels (red), a hinged connection between the 2 halves (brown), a frame (blue), a regulable acoustic adhesion system (green), and a chip/shell (black). (B) Schematic of acoustic adhesion generation. The disk vibrations, driven by a vibration motor, periodically expel and intake air. A negatively pressured air layer is formed beneath the vibration disk as more air is gradually squeezed out, leading to anisotropic adhesion with strong normal vacuum strength (~1 kPa) and weak tangential friction, which is extremely low and even negligible. (C) The robot traversed through a 10-mm step, a vertical wall, and an inverted ceiling and finally found a crack sign on an engine blade. The wireless-controlled robot is capable of carrying a 70-g weight and driving at 70 mm/s on both vertical and inverted surfaces. (D) Operation logic diagram of an AI-integrated robot. Ambiguous voice commands were transformed into precise robot actions, simplifying the control process.

In this study, we designed a wheeled climbing robot (20 g in total weight), comprising of 2 adhesion units (52 mm × 57 mm × 27 mm in size), as shown in Fig. 1A. The components are classified into 4 color-coded: acoustic adhesion parts (green), driving system (pink), rotating joint (brown), and roller components (blue). The adhesion force is controlled by switching the vibrating motor mounted on a flexible disk on or off. The air layer between the disk and substrate directly affects the nature and magnitude of the adhesive force. Thus, we implemented a linear servo-spring to regulate the adhesion force. Four motorized rubber wheels drive the robot to move straight or turn with a zero radius. A rotating joint servo was designed to connect 2 modular adhesion units, which enables climbing over corners and stairs. Two rollers on the robot frame provide extra support torque when traversing uneven surfaces, such as gaps, stairs, and corners. The robot is equipped with an ESP32 chip and a motor control chip, enabling AI-driven interactive intelligence through voice recognition and task planning. A more detailed internal structure diagram and the linear servo-spring system are provided in Fig. S1.

As the core component of wall-climbing robots, the acoustic adhesion disk with an embedded vibration motor generates a stable negative pressure cavity for adhesion to the surface (Fig. 1B). Meanwhile, the air layer between the disk and the substrate has minimal tangential friction, enabling easy lateral driving by the motorized rubber wheels. The tunable anisotropic acoustic adhesion system and rotating joint allow enhanced adhesion strength and rapid climbing on continuous flat/curved surfaces, as well as nonuniform gaps, corners, and stairs. For example, the dual-unit climbing robot can navigate up a step and then ascend a vertical wall (Fig. 1C and Movie S1). Specifically, to climb onto a vertical wall, the front unit is lifted as the rear unit maintains adhesion to the floor. The rear part adjusts its position and angle relative to the front part, allowing the front to attach to the wall. The rear part detaches when its vibrating motor is powered off, and the front part climbs up until the rear unit rotates and connects to the wall. The robot can carry a 70-g payload and drive at 70 mm/s on both walls and ceilings (as shown in Fig. 1C, inset). The 3D-printed chassis integrates all robot components for easy assembly. An onboard camera enables the robot to detect obstacles and search for targets, as demonstrated by its successful identification of a crack sign on an aero-engine blade (Fig. 1C). The robot utilizes multiple microcontrollers and wireless communication systems to guide its posture and movements precisely.

Obstacle-traversing expands a robot’s motion and detection scope from continuous surfaces to a 3D domain. While this expanded range enhances operational capability, it also complicates the planning and control of drive motors, vibrating motors, and positional servos. Recent advances in voice intelligence and large language models (LLMs) offer a streamlined solution to this challenge (Fig. 1D). We preconfigure the LLM with essential robot parameters (Text S1), enabling it to interpret human voice commands received via an onboard microphone and Wi-Fi module. These commands are converted to text through cloud-based speech recognition and subsequently processed by an online LLM (e.g., ChatGPT 4, OpenAI, USA), which decomposes them into specific executable actions (Fig. 1C and Movie S1). Operators can further adjust the process via a smartphone-based graphical interface, while real-time operational status and sensor data are displayed for monitoring. By integrating voice recognition and task planning, this approach greatly simplifies complex robotic motion algorithms and control.

### Anisotropic acoustic adhesion

**Fig. 2.**
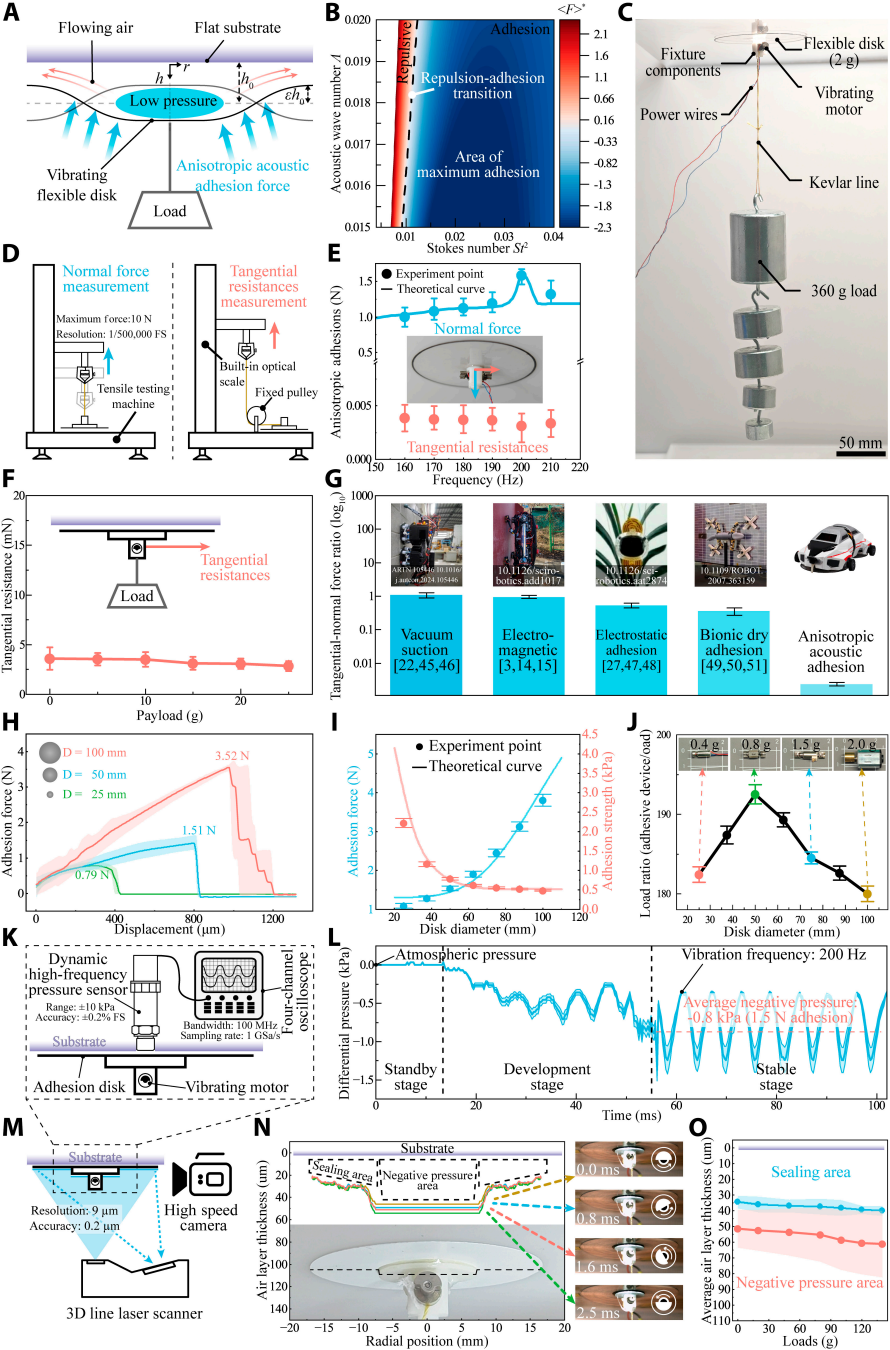
The generation and performance of anisotropic acoustic adhesion. (A) A schematic diagram illustrates the generation of acoustic adhesion resulting from dynamic vibrations. The disk periodically squeezes more air out than it inhales, given proper disk dimensions and vibration frequencies, leading to a negative pressure cavity relative to ambient pressure. (B) Relationship between dimensionless adhesion force ${\langle F\rangle }^{*}$ with the Stokes number $\alpha^{2}$ and acoustic wave number Λ. Under specific threshold conditions, adhesion and repulsion forces undergo role reversal, a phenomenon primarily governed by gap thickness. This behavior is consistent with fundamental physical principles: When the Stokes number falls below a critical value (indicating an excessively narrow gap), particle collisions dominate, thereby generating repulsive forces. (C) An acoustic adhesion disk (100 mm in diameter) triggered by a 0.12-W vibration motor successfully held a 360-g load on inverted ceiling surfaces, which is 180 times the disk’s 2-g weight. (D) Schematic diagram of normal adhesion and tangential resistance measurement. The tensile tester incorporates an integrated linear encoder and strain gauge force transducer, enabling convenient measurement of the adhesion disk’s normal adhesion force. For measuring tangential resistance, we designed and fabricated a fixed pulley system. By routing Kevlar cord through the fixed pulley to alter the measurement direction, tangential resistance can be accurately assessed. (E) Anisotropic acoustic adhesions indicate strong normal adhesion but extremely low tangential resistance, which is highly advantageous for the design of wheeled climbing robots. (F) Relationship between tangential force and load variation. (G) Comparison of tangential to normal force ratio between various adhesion mechanisms [3,14,15,22,27,45–51]. Specifically, anisotropic acoustic adsorption demonstrates significantly reduced tangential resistance, thereby enabling the fundamental basis for high-speed wheeled locomotion systems. (H) The normal adhesion force with various disk diameters was measured by tensile testing, where the disk was pulled away from the substrate. A larger disk size helps enhance adhesion forces. (I) The adhesion force and strength change with various disk diameters. The decline in adhesion strength is primarily attributed to the adhesion area increasing at a faster rate than the adhesion force can sustain. The theoretical curve is shown alongside the experimental data points. Their close congruence validates the reliability of the proposed theoretical model. (J) Load ratio curve chart for adhesive devices with different disk diameters paired with corresponding vibration motors. Since larger disks necessitate stronger vibration sources for excitation, consuming increased adhesive force, the load ratio declines. Consequently, the 50-mm disk was selected to maximize load efficiency. (K) Schematic of the setup used to measure pressure variations within the air layer of the adhesive disk. (L) Temporal pressure profile in the adhesive disk air layer, where the *y* axis indicates the pressure difference relative to atmospheric pressure. (M) Schematic diagram of the thickness measurement of a thin air layer under disks using a 3D line laser scanner and a high-speed camera. (N) The thickness of the air layer dynamically distributes during half a vibration cycle. The air layer can be divided into 2 distinct regions: the sealing area at the edge and the negative pressure area at the center of the disk. (O) Influence of payloads on average air layer thickness at disk center and edge sections.

Acoustic adhesion is generated and controlled by a vibration motor; periodic deformations of a flexible annular disk trigger dynamic airflow beneath the disk. We observed normal adhesions with proper disk materials and vibration parameters and assumed that a negative pressure differential was generated relative to the ambient atmosphere (Fig. 2A).

We developed a theoretical model based on the traditional squeeze-film adhesion theory, as outlined below. The thickness of the air layer *h* between the disk and the substrate can be described as $h\left(r,\;t\right)$, a function of the distance from the center of the disk *r* and time *t*. The periodic oscillation of the air layer thickness can be expressed as:

$$h\left(r,\;t\right)=h_{o}\left[1+\varepsilon S\left(r/R\right)\text{cos}\left(\omega ,t\right)\right]$$ (1)

where $h_{0}$ is the average gap thickness, εis the amplitude of oscillation, *R* is the disk radius (25 mm in this study), and ω is the angular frequency. The peak amplitude profile $S\left(r/R\right)$ depends on annular disk thickness, stiffness, and vibration frequency.

The acoustic adhesion force due to the negative pressure is obtained by integrating the pressure difference over the area of the adhesion disk:

$$F_{\mathrm{ad}}=\int_{0}^{R}\left(p\left(r,\;t\right)-p_{a}\right)2\pi rdr$$ (2)

Here, $p_{a}$ denotes the atmospheric pressure, set at 0.1 MPa in this study. The pressure at different radii $p\left(r,\;t\right)$ is primarily determined by $h\left(r,\;t\right)$ [36–38]. Therefore, the adhesion force is cyclic; for simplicity, all subsequent references to adhesion force refer to the time-averaged value unless otherwise specified. These equations suggest that the adhesion force should depend on the vibration frequency ω, annular disk radius R, and air layer thickness $h_{0}$.

Hydrodynamic problems involving multiple coupled parameters are often complex to solve, so we use dimensionless numbers to simplify multiple influencing parameters and assess critical parameters. The relationship between the actual adhesion force $F_{\mathrm{ad}}$ and dimensionless adhesion force ${\langle F\rangle }^{*}$ is:

$$F_{\mathrm{ad}}=\varepsilon^{2}\Lambda p_{a}\pi R^{2}{\langle F\rangle }^{*}$$ (3)

The dimensionless adhesion force ${\langle F\rangle }^{*}$ in a steady state consists of an inner force contribution ${\langle F_{i}\rangle }^{*}$ and an edge force ${\langle F_{e}\rangle }^{*}$. Since the edge force ${\langle F_{e}\rangle }^{*}$ accounts for 0.5% of the total force ${\langle F\rangle }^{*}$, the contribution of the edge force is negligible, and hence, the total dimensionless force can be simplified as:

$${\langle F\rangle }^{*}\left({\mathrm{St}}^{2},\;\Lambda \right)∼\frac{Z_{1}\Lambda }{\alpha^{2}}+Z_{2}\Lambda +Z_{3}$$ (4)

where ${\mathrm{St}}^{2}$ is the Stokes number, which quantifies the relative importance of viscous diffusion (i.e., ${\mathrm{St}}^{2}$ is inversely proportional to the fluid viscosity). Λ is the acoustic wave number, which quantifies the effects of fluid compressibility (i.e., the smaller Λ, the less compressible the fluid is). $Z_{1∼3}$ denote auxiliary content involving Bessel functions arising from the disk geometry and vibration waveform (see Text S2 for details). The expressions for the Stokes number ${\mathrm{St}}^{2}$ and the acoustic wave number Λ are as follows:

$${\mathrm{St}}^{2}=\frac{{h_{0}}^{2}\omega }{u_{a}/\rho_{a}}$$ (5)

$$\Lambda =\frac{R^{2}\omega^{2}}{p_{a}/\rho_{a}}$$ (6)

where $u_{a}$ is the aerodynamic viscosity (0.018 mPa·s, 20 °C) and $\rho_{a}$ is the air density (1.2 kg/m^3^). By adjusting the dimensionless parameters of ${\mathrm{St}}^{2}$ and Λ, a transition from levitation to adhesion can be achieved, as shown in Fig. 2B. This implies that acoustic adhesion can be precisely controlled by adjusting the thickness of the air layer. This theoretical finding aligns with physical intuition: When a vibrating disk is placed too close to the substrate, repulsive forces arise due to collisions and gas compression—a phenomenon known in industrial settings as acoustic suspension. This consistency provides preliminary validation of the model’s plausibility. To demonstrate the load performance, a 360-g weight was hung on a 100-mm-diameter adhesion disk (Fig. 2C and Movie S2), indicating a load ratio of 180 over the disk’s 2-g weight. High load ratios (roughly 5 to 50) of adhesion were reported [18,27,28,44], but less than that in this work. To further assess its accuracy, we analyzed the influence of 2 key parameters—vibration frequency and film radius—on the adhesion force and compared the theoretical predictions with experimental measurements as follows.

The vibration frequency is a critical parameter, and we used a tensile testing machine to characterize the normal adhesion force and tangential resistance of the acoustic adhesion on 50-mm-diameter disks, shown in Fig. 2D. The frequencies were adjusted by changing the voltages on the vibration motor (Fig. S2). Since a low frequency cannot excite the disk to deform, a proper disk vibration frequency of 150 to 210 Hz is set up to generate 1.5-N normal adhesion force in Fig. 2E. Experimental data are plotted as discrete points, whereas theoretical predictions are illustrated by continuous curves. The close alignment between the 2 sets of results demonstrates agreement, thereby affirming the validity of the theoretical model. A peak response is observed at 200 Hz, as this frequency lies in the vicinity of the resonant frequency of the disk. The resulting resonance amplifies the disk’s vibration, leading to more efficient expulsion of air from the cavity and the generation of a stronger negative pressure. We confirm that anisotropic acoustic adhesion generates low tangential force, measured to be less than 1% of the normal force, due to the thin air layer acting as an air cushion. This makes acoustic adhesion ideal for designing wheeled climbing robots that require strong normal adhesion but low tangential resistance.

We investigated the tangential resistance of adhesive disks under varying normal loads in Fig. 2F. Our results demonstrate a consistent decrease in tangential resistance with increasing load. This observation aligns with the physical experience for acoustic adsorption disks, wherein the resistance originates from transient contact events between localized regions of the disk and the substrate during vibration. Higher loads increase the thickness of the intervening air layer, thereby reducing the probability of these transient contacts and consequently diminishing tangential resistance. Crucially, however, the tangential resistance magnitude is negligible—typically orders of magnitude smaller—compared to the normal adhesive force across all tested conditions.

The remarkably low tangential resistance exhibited by anisotropic acoustic adhesion confers substantial advantages over conventional adhesion mechanisms. By eliminating the reliance of adhesive methods on legged robots, this characteristic further enables deployment on wheeled platforms, thereby enhancing mobility efficiency. The tangential-to-normal force ratios were analyzed and compared across various adhesion mechanisms as illustrated in Fig. 2G. This ratio quantifies the tangential force required per unit of normal adhesive force. Analysis reveals that existing adhesion methods consistently exhibit tangential-to-normal force ratios of about 1 (the axes in the figure have been plotted using a log_10_ scale). This ratio signifies that the magnitude of tangential resistance is comparable to the normal force (the data for vacuum suction were obtained from [22,45,46], electromagnetic adhesion from [3,14,15], electrostatic adhesion from [27,47,48], and biomimetic dry adhesion from [49–51]). Consequently, wall-climbing robots utilizing these conventional adhesion methods predominantly employ legged or wheel-legged locomotion schemes. These schemes necessitate the complete detachment of the adhesion mechanism during movement, which inherently imposes a marked constraint on robot speed. In contrast, the anisotropic adhesion mechanism proposed herein demonstrates an exceptionally low tangential-to-normal force ratio. This characteristic implies minimal drag resistance during locomotion, even while maintaining continuous adhesion. Consequently, this enables the realization of wheeled wall-climbing robots, substantially enhancing their mobility efficiency.

Although some research teams have incorporated structural designs enabling applications such as negative pressure [9,52] and dry adhesion [53] in wheel-legged robots, they demonstrate strong adhesion performance yet face inherent challenges in balancing structural compactness with strong adhesion force. Negative pressure-based approaches require complex vacuum units that substantially compromise miniaturization and mobility efficiency [9,52]. Micro-suction wheel designs require preloading, which constrains autonomous locomotion, particularly on ceilings [53].

The effect of disk diameter on adhesion force was quantified, as shown in Fig. 2H. Normal adhesion force was characterized by pulling the vibrational disk with various diameters (25, 50, and 100 mm). The maximum adhesion force increased with diameter, as did the displacement during detachment. Note that the areas under the curves represent the adhesion energy during pulling, which are 0.26, 0.8, and 2.3 mJ, corresponding to the diameters of 25, 50, and 100 mm, respectively.

The adhesion strength and force exhibit opposite trends with increasing disk diameter in Fig. 2I. This occurs primarily because increasing the adhesive disk diameter enhances the contact area more significantly than the adhesive force, consequently leading to a reduction in adhesive pressure. The theoretical curve is shown alongside the experimental data points. Their close congruence validates the reliability of the proposed theoretical model.

Critically, larger diameters necessitate higher vibration excitation energy. Compact vibration motors cannot induce optimal amplitudes in large-diameter membranes, requiring heavier motors that consume additional adhesion force for self-actuation rather than payload lifting. We quantified this trade-off through the load ratio metric (payload-to-adhesion device mass ratio) in Fig. 2J, revealing that disks with ~50 mm diameter achieves peak efficiency (adhesion device mass: 0.8 g; payload: 154 g; load ratio: 192.5).

The fundamental mechanism underlying anisotropic acoustic adsorption is attributed to negative pressure. To investigate this, we employed a dynamic high-frequency pressure sensor coupled with an oscilloscope to monitor pressure variations inside the negative pressure cavity, as illustrated in Fig. 2K. The complete pressure profile across the gap between the vibrating disk and the substrate was captured, as shown in Fig. 2L. The *y* axis indicates the pressure difference relative to atmospheric pressure. The recorded curve can be categorized into 3 distinct phases: the standby phase, during which the vibration motor remains inactive; the development stage, where the disk oscillates under motor actuation, expelling more gas than it intakes, thereby progressively forming the negative pressure cavity; and the stable stage, in which the load and adhesive forces reach equilibrium. During this final phase, the internal negative pressure oscillates at the motor’s vibration frequency of 200 Hz, stabilizing at an average value of –0.8 kPa. Given a disk diameter of 50 mm, this negative pressure corresponds to an adhesive force about 1.5 N, a result consistent with previously reported experimental measurements.

The anisotropic behavior of near-field acoustic adhesion arises from the presence of an air layer between the annular disk and the substrate. This thin layer of air with negative pressure not only generates strong adhesions along the normal direction but also leads to ultralow tangential friction due to the air cushion. Prior theoretical work has confirmed the critical role of air layer sensitivity [54]. In this paper, the critical air layer and disk vibration frequency are precisely quantified by a 3D line laser scanner and a high-speed camera (Fig. 2M).

We measured the air layer thickness distribution during half a repeated vibration cycle, as illustrated in Fig. 2N and Movie S2. The vibration motor is glued to a rigid support, so the center of the flexible disk cannot fluctuate as the edge part does. Negative pressure is formed and accumulated at the disk center within an air layer thickness of ~50 μm, and the flexible edge fluctuates as a sealing area (~20 μm). Four curves in Fig. 2N indicate continuously increasing thicknesses of the center disk in half a vibration cycle, measured by a 3D line laser scanner. A high-speed camera synchronously captures 4 frames of the sector eccentric wheel on the vibration motor (Fig. 2N), indicating a vibration frequency of 198 Hz. Notably, the air layer thickness in the sealing area exhibits minimal variation during vibrations compared to that at the disk center. This phenomenon is primarily attributed to the Bernoulli effect: The Bernoulli force suppresses the flexible disk edge as airflow passes through the narrow slit beneath the disk edges.

We further investigated the behavior of the air layer under various loads, as shown in Fig. 2O. As the load increases from 0 to 140 g, the average air layer thickness in the sealing area changes slightly, as indicated by the blue solid line. Meanwhile, the vibration amplitude in this area remains nearly constant, as shown by the blue-shaded area in Fig. 2O. In contrast, the average thickness in the negative pressure area increases more significantly, and the vibration amplitude becomes substantially larger with higher loads, indicated by the red solid line. As the average air layer thickness increases, the available space for compression also increases, thereby enhancing the adhesion force to sustain heavier loads.

Measurements of air layer pressure and thickness validate our theory: Under periodic external forces, the flexible annular disk undergoes periodic oscillations. The persistent air layer between the disk and substrate, which accounts for the observed small tangential force, enables external air leakage into the cavity through this gap. Consequently, the volume of gas expelled per cycle exceeds that drawn in due to the disk’s deformation dynamics. This net outflow effectively compensates for leakage losses, gradually establishing a stable negative pressure zone between the substrate and the disk. As experimentally demonstrated (Fig. 2C), this mechanism generates marked adhesive forces.

### Active adhesion control system and resonance optimization

**Fig. 3.**
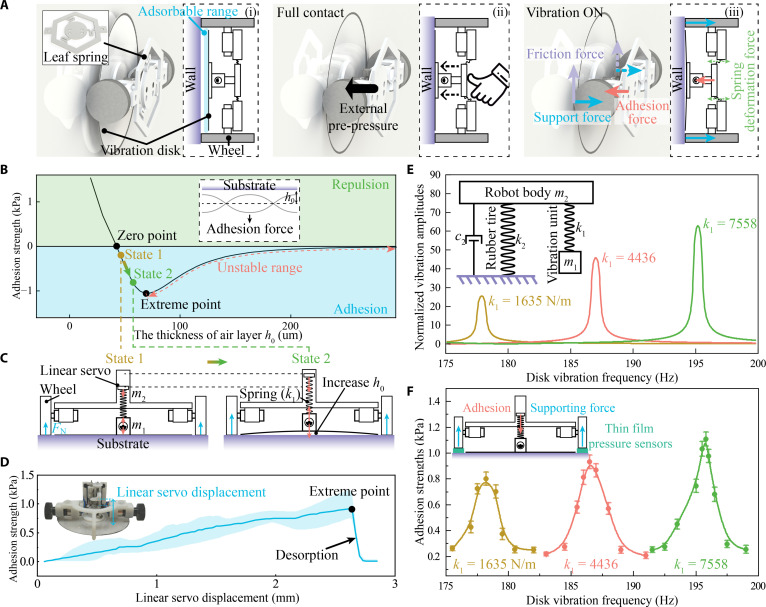
Principle of active control and resonance analysis. (A) Schematic diagram of the manual initialization process of the existing wall-climbing robot with leaf-spring structure. (B) Influence of air layer thickness (the average distance $h_{0}$ when the disk vibrated) on adhesion force. With the increase in air layer thickness, the force transitions from repulsive force (blue area) to an attractive force (green area) and reaches its maximum, after which it loses stability, causing detachment failures. Adhesion can be regulated by controlling the air layer thickness between the zero point and the extreme point. (C) A linear servo-spring system was designed to control the air layer actively. The linear system also transmits adhesion force to the robot body and provides wheel pressure for vertical climbing. (D) Adhesion strength continuously increases as the servo-spring system is lifted, confirming the effectiveness of the active air layer regulation system. The air layer changes only slightly due to the adaptive compensation of the springs. (E) Resonance model and analysis of the robot. The resonance model includes robot body mass $m_{2}$ and vibration unit mass $m_{1}$ (including disk and motor), with coefficients of $k_{2}$ and $k_{1}$, respectively. The theoretical calculations of normalized vibration amplitudes indicate the influence of vibration frequency on different spring stiffness $k_{1}$. The amplitudes resonate and increase dramatically at distinct disk vibration frequencies, regarded as resonance frequencies. (F) The adhesion strength, equivalent to supporting force on tires, is measured using a thin-film pressure sensor. Distinct peak values of adhesion occur at resonance frequencies with various springs $k_{1}$, as agreed upon by theoretical analysis.

Based on the previous analysis, acoustic adhesion relies on the edge-sealing effect of the flexible disk, which requires the adhesion disk to be close to the substrate surface. When the disk is directly mounted on leaf springs, the substrate is not within the range of adsorbable distances (Fig. 3Ai). External pre-pressure is necessary to manually press down leaf springs (Fig. 3Aii), ensuring that contacted surfaces initiate adhesion. Once the vibration is stable, the adhesion force is converted through the deformation of the leaf springs into a supporting force on the robot wheels, generating friction to support the robot and load as it drives on walls (Fig. 3Aiii). The stable adhesion indicates anisotropic performance with the formation of a thin air layer beneath the disk. Close contact between the disk and substrate is only necessary at the initial stage of adhesion generation, and constant contact can cause severe wear.

Thus, the implementation of an active adhesion control system (recall the adhesion unit in Fig. 1A) is essential for robots to perform complex maneuvers, including autonomously traversing uneven corners and gaps. During horizontal locomotion, the system lifts the disk to reduce frictional resistance and improve mobility. To engage adhesion, the system lowers the disk to establish firm contact with the substrate and then retracts the disk when adhesion is stable. The vertical displacement of retraction directly affects the generated adhesion forces.

Utilizing the acoustic adhesion model described previously, the relationship between adhesion force and air gap thickness, presented in Fig. 3B, is calculated. This relationship is also evidenced in Fig. 2B: For any given vertical coordinate, as the Stokes number ${\mathrm{St}}^{2}=\rho_{a}{h_{0}}^{2}\omega /u_{a}$ increases, the adhesive force transitions from repulsion to attraction, followed by a gradual decrease.

Theoretical calculations confirm the adhesion principle of a thin air layer and point out a peak adhesion strength with a proper air layer. The repulsion–attraction transition occurs at a small thickness of the air layer $h_{0}$, roughly 30 μm (as the zero point in Fig. 3B). While the force is repulsive as the air layer $h_{0}$ is less than the zero point, the force becomes attractive beyond this boundary. Optimized thickness led to a maximum adhesion peak, labeled as the extreme point. Negative pressure peak values were also reported based on theoretical predictions with various substrate materials and disk dimensions [54]. Beyond the extreme point, the adhesion decreases irreversibly (unstable range in Fig. 3B), resulting in sudden detachments. Therefore, achieving stable adhesion requires a strict thickness range of the air layer between the zero point and the extreme point, and loads should also be less than the peak adhesion on inverted ceilings or the maximum friction on vertical walls.

Since the effective air layer is limited to a small range of roughly 60 μm [37,38], it is necessary to carefully control the air thicknesses. To achieve strong adhesions by regulating the air layer, a linear servo-spring system was accordingly designed in this work (Fig. 3C). A linear servo adjusts the air layer (like a course adjustment) and a mounted coil spring compensates for the servo displacement error (like a fine adjustment) because the forced disk vibration tends to stabilize at a distinct air layer thickness. The linear servo integrates a control chip internally. Upon receiving commands, it drives the motor-lead screw mechanism to achieve linear motion. The moving component is connected to a sliding resistor, which provides real-time position feedback to the control chip through resistance changes. The chip automatically compensates for positional errors, and the integration with adaptive spring buffering enables precise real-time adjustment of the air gap dynamics.

Given a fixed frequency of the vibration motor, the disk vibration frequency and air layer thickness are determined to generate adhesions. Specifically, the servo-spring system slightly lifts the disk upon contact with substrate and transmits the adhesion force from the vibrating disk to the robot body, generating normal pressure on the wheels as state 1 in Fig. 3C. As the servo further lifts the disk, the coil spring is stretched to promise a distinct air thickness for stable adhesion vibrations, achieving precise distance control, thereby enhancing adhesion as state 2 in Fig. 3C. In contrast to robots that rely solely on leaf springs as a connecting mechanism [36,38]—which need manual pre-pressure to make the adsorption structure adhere to the substrate—this system allows for fully autonomous, smooth adjustment of the adhesion force according to the substrate material and load, thereby fully exploiting the adhesion. Higher normal adhesion enables the robot to carry more loads on inverted surfaces, and it also works on vertical walls, considering that higher normal adhesion can provide more tangential friction via rubber wheels. To verify the effectiveness of this active adhesion control system, we characterized it with a thin-film resistive pressure gauge. Increasing the displacement of the servo effectively enhances adhesion strength, reaching a peak value (Fig. 3D).

Although a coil spring helps adjust the acoustic adhesion, it can cause irregular vibration of the robot’s body through resonance. Thus, the mechanical resonance of the vibrational disk and robot was analyzed and balanced to ensure disk vibration and adhesion performance. The masses of the acoustic adhesion disk and robot body are $m_{1}$ and $m_{2}$, and corresponding displacements are $x_{1}$ and $x_{2}$, respectively (inserts in Fig. 3E). The wheels on solid substrates are regarded as a spring damping system with coefficient $k_{2}$ and damping factor $c_{2}$. The coefficient of the coil spring is $k_{1}$. The force balance equation is accordingly proposed as follows:

$$M\ddot{x}+C\dot{x}+\mathrm{Kx}=F$$ (7)

where M, C, K, and x are the quality matrix, damping matrix, stiffness matrix, and displacement matrix, respectively (details in Text S3). The centrifugal force of the eccentric wheel on vibration motors and the adhesion force are large and crucial as driving inputs to the system. The dynamic adhesion force indicates a matched frequency with that of the vibration motor. This characteristic enables us to combine the effects of both forces into a unified periodic force for further analysis, $F=\left({\bar{F}}_{\mathrm{ad}}+{\bar{F}}_{\mathrm{vib}}\right)\text{sin}\left(\omega ,t\right)$, where ${\bar{F}}_{\mathrm{ad}}$ is the adhesion force and ${\bar{F}}_{\mathrm{vib}}$ is the vibrating force. We exclude gravity from the external force *F* because static forces do not affect the vibration amplitude or frequency, only the equilibrium position. The disk vibration amplitude $A_{1}$ is deduced by solving the above equations, and the dimensionless expression is:

$$\frac{A_{1}}{x_{\mathrm{st}1}}=\sqrt{\frac{{\left(\beta^{-1}+\delta^{2}-\lambda^{2}\right)}^{2}+4\zeta^{2}\delta^{2}\lambda^{2}}{{\left(\lambda^{2}\beta^{-1}-\left(1-\lambda^{2}\right)\left(\delta^{2}-\lambda^{2}\right)\right)}^{2}+4\zeta^{2}\delta^{2}\lambda^{2}{\left(1-\lambda^{2}\right)}^{2}}}$$ (8)

In this formula, the symbol $x_{\mathrm{st}1}=A_{0}/k_{1}$ denotes the static deformation of the disk ($A_{0}$ corresponds to the external force at a frequency of zero); $\beta =m_{2}/m_{1}$ represents the mass ratio; $\delta =\omega_{a}/\omega_{0}$ indicates the ratio of natural frequencies; $\zeta =c_{2}/2m_{2}\omega_{a}$ corresponds to the damping ratio; $\lambda =\omega /\omega_{0}$ refers to the excitation frequency ratio; ${\omega_{a}}^{2}=k_{2}/m_{2}$ signifies the natural frequencies of the robot; and ${\omega_{0}}^{2}=k_{1}/m_{1}$ stands for the natural frequencies of the disk. The amplitude $A_{1}$ indicates dependence on the spring coefficient $k_{1}$ and dependence ω, normalized to rated frequency *λ*. Therefore, optimal acoustic adhesion can be achieved with an appropriate spring coefficient and regulated disk frequency using vibration motors.

According to theoretical models, the normalized vibration amplitude ratio $A_{1}/x_{\mathrm{st}1}$ increases sharply at specific disk vibration frequencies (Fig. 3E), indicating that resonance has occurred. The enhanced vibrations can be repeatedly observed, given a different spring coefficient $k_{1}$. Typically, resonance should be avoided as it can cause potential machine damage. Here, on the contrary, we trigger the disk to resonate directly, thereby enhancing the adhesion force [36–38,54]. Adhesion strength is measured using a thin-film pressure sensor (inset of Fig. 3F), where the support force exerted on the tires is converted into adhesion strength according to the principle of force equilibrium. The experimental results of adhesion strength, as shown in Fig. 3F, align well with normalized amplitude calculations (Fig. 3E) at various disk frequencies *λ*, leading to resonance optimization.

### Robot dimension optimization and stability analysis

**Fig. 4.**
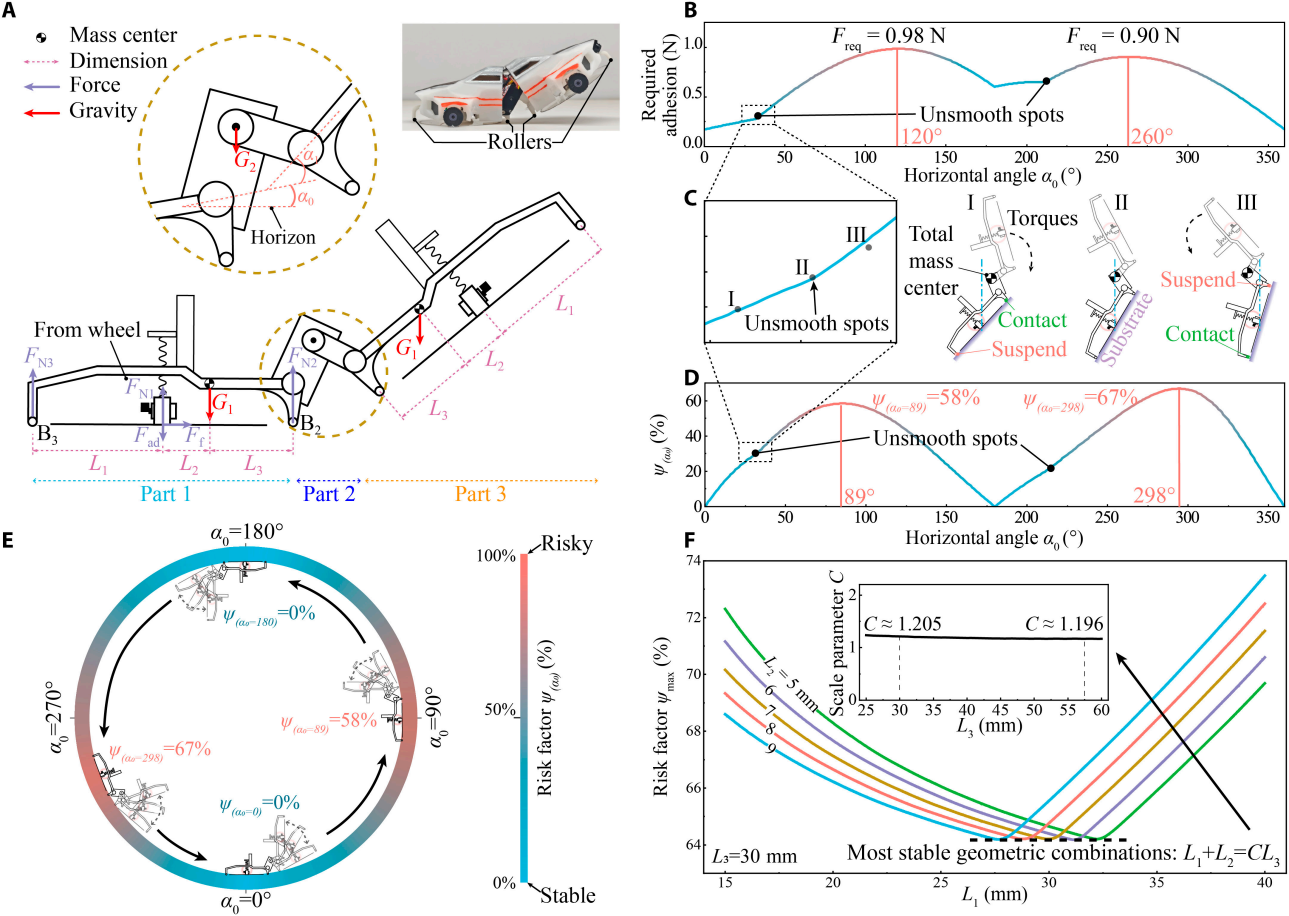
The stability analysis and dimension design of climbing robots. (A) Mechanical model of the robot and its geometric, rotation, and gravity parameters. (B) Quantitative correlation between the adhesion force required for stable interfacial transition of the wall-climbing robot and the initial contact angle. (C) Two unsmooth spots correspond to switches in the working state of 2 rollers. Before the gravity center aligns with the adhesion center, the gravity moment is clockwise, which allows only the upper roller to contact the substrate (green) but not the lower roller (red), and vice versa after alignment. (D) The defined risk factor $\psi_{\left(\alpha_{0}\right)}$ changes with the original angle $\alpha_{0}$ and represents 2 dangerous positions at peaks. (E) The climbing stability analysis assumes that the robot lifts the front part at various positions inside the cylinder. The safest states are at 0° and 180°, while the riskiest angles are 89° or 298° due to the shift in the gravity center. (F) Optimal analysis of robot dimensions. $L_{1}$, $L_{2}$, and $L_{3}$ all affect the climbing stability since they are the force arms of gravity moment. Curves with various $L_{2}$ consistently point out the most stable states when $L_{1}+L_{2}=1.2L_{3}$, leading to a safety design guide.

The anisotropic adhesion provides a strong adhesion force, enabling the robot to climb on continuous, flat, and curved surfaces. However, a single robot unit is unable to traverse stairs or cross corners. A modular robot with 2 adhesion units was demonstrated to solve this issue (recalling Fig. 1C). Still, it experiences a relatively large gravity moment for the adhesion disk, resulting in adhesion failures. We established a robot balance model to quantify the stability boundary conditions for wall-climbing locomotion. Based on this analysis, we integrated 2 supporting rollers at the robot’s periphery to generate counteracting moments. This design enhances the moment equilibrium of the adhesion disk, thereby preventing detachment under operational loads. The diagram structure of the entire robot is also presented in Fig. 4A. The rear adhesion unit (part 1) adheres to the substrate, and a surface-traversing servo (part 2) lifts the front part (part 3) to a total angle of $\alpha_{1}$ + $\alpha_{0}$. Only one side roller serves as a supporting point (for example, $B_{2}$ in Fig. 4A), struts on the ground, and the adhesion force (part 1) provides counter-torque against the gravitational moment of the lifted front part (part 3). The left roller ($B_{3}$) wheel is off the ground, similar to a teeter-totter, and provides no support.

We used coordinate transformation methods to analyze force and moment balance (details in Text S4 and Fig. S3):

$$\begin{array}{c} \begin{array}{l} \left\{\begin{array}{c} F_{N2}\left(L_{1}+L_{2}\right)+M_{G}=0,F_{N3}=0\;\text{when}\;M_{G}<0,\\ F_{N3}L_{3}+M_{G}=0,\;F_{N2}=0\;\text{when}\;M_{G}>0, \end{array}\right.\\ G_{x}=F_{f}\\ G_{y}=F_{N1}+F_{N2}+F_{N3}-F_{\mathrm{ad}} \end{array} \end{array}$$ (9)

where $F_{N1}$, $F_{N2}$, and $F_{N3}$ mean the supporting forces on wheels, roller $B_{2}$, and roller $B_{3}$, respectively. These $L_{x}\left(x=1,\;2,\;3\right)$ are dimension parameters in Fig. 4A. The gravity moment $M_{G}$ determines the working state of 2 rollers. Specifically, the right roller $B_{2}$ provides support moment, and the left roller $B_{3}$ slightly suspends when $M_{G}<0$, and vice versa when $M_{G}>0$.

Robotic failure modes primarily comprise insufficient adhesion force (leading to falls or overturns) and insufficient tire friction. During normal locomotion, the concurrent operation of both adhesive devices substantially enhances safety relative to scenarios involving single adhesion device functionality across interfaces. Consequently, this investigation prioritizes the more hazardous surface-traversing conditions. To address scenarios involving insufficient adhesion force, we leverage the static equilibrium equations established previously and posit that an active control system can generate a precise support force at the wheels, thereby inducing adequate friction for stability. Under this assumption, the requisite adhesion force $F_{\mathrm{ad}}$ for the wall-climbing robot is derived as:

$$\left\{\begin{array}{cc} F_{\mathrm{ad}}=\frac{G_{x}}{\mu }+\frac{-M_{G}}{\left(L_{1}+L_{2}\right)}-G_{y} & \text{when}\;M_{G}<0,\\ F_{\mathrm{ad}}=\frac{G_{x}}{\mu }+\frac{-M_{G}}{L_{3}}-G_{y} & \text{when}\;M_{G}>0, \end{array}\right.$$ (10)

Fixing the initial contact angle $\alpha_{0}$, we define the maximum adhesion force required across all servo angles $\alpha_{1}$ as the critical adhesion force for that initial angle. The computed critical adhesion forces for varying initial angles are presented in Fig. 4B. The results demonstrate that larger adhesion forces are necessary at angles 120° and 260°. This requirement arises because the gravitational lever arm acting on the robot’s front-mounted components is maximized during the surface traversing at these specific angles. Moreover, the unsmooth spots (Fig. 4B and C) are found when the mass center aligns with the adhesion disk. Different initial angles $\alpha_{0}$ change the direction of gravity moment $M_{G}$ and the working state of rollers in Fig. 4C. As a result, the force balance suddenly changes and causes unsmooth points on the curve.

According to the anisotropies of acoustic adhesions, normal adhesions $F_{\mathrm{ad}}$ help the climbing robot carry loadings on ceilings. Regarding the vertical walls, normal adhesions $F_{\mathrm{ad}}$ generate friction on the wheels, providing resistance against gravity. Compared to adhesive failure, friction failure is more common in wheeled robots (since it requires multiplying by the coefficient of friction). Therefore, the maximum friction $F_{f\max}$ on walls governs the payload,

$$\begin{array}{c} F_{f\text{max}}=\mu F_{N1}=\left\{\begin{array}{c} \mu \left(G_{y}+F_{\mathrm{ad}}+\frac{M_{G}}{L_{1}+L_{2}}\right)\;\text{when}\;M_{G}<0\\ \mu \left(G_{y}+F_{\mathrm{ad}}+\frac{M_{G}}{L_{3}}\right)\;\text{when}\;M_{G}>0 \end{array}\right. \end{array}$$ (11)

For a safe climbing case, the required friction $F_{f}$ on walls has to be less than maximum static friction $F_{f\max}$. Thus, we defined the risk factor *ψ* to quantify the stability of climbing:

$$\psi_{\left(\alpha_{0},\;\alpha_{1}\right)}=\frac{F_{f}}{F_{f_{\max}}}$$ (12)

*ψ* specifies the possibility of robot detaching, and smaller *ψ* implies stabler motion. Climbing on walls can be safe if *ψ* is less than 70% according to normal engineering safety requirements [55].

The risk factor *ψ* is relative to 2 angles of tilted substrate angle $\alpha_{0}$ (0° for horizontal surface) and joint angle $\alpha_{1}$ (Fig. 4A). Assuming that the robot climbs inside a cylindrical tube, and the rear adhesion unit (part 1) always adheres to the tube, the joint angle $\alpha_{1}$ changes with the range of 0° to 100° (Fig. 4D and E). We defined $\psi_{\left(\alpha_{0}\right)}={\text{max}}_{\alpha_{1}\in \left[0,100\right]}\left(\psi_{\left(\alpha_{0},\alpha_{1}\right)}\right)$ to characterize the danger of the robot when lifting the other half of the fuselage at $\alpha_{0}$ angle. The riskiest points emerge when tilted substrate angle $\alpha_{0}$ is at 89° or 298° because the total mass center is furthest away from the robot, causing the largest gravity moment. It differs from typical exceptions of 135° and 225° since the gravity center rarely coincides with the dimension center during climbing. This contrasts with the most critical point identified in Fig. 4B, which deals with conditions of insufficient adhesion, while the present case relates to situations involving inadequate wheel friction.

The lever arms can be adjusted to limit gravity moment and improve the climbing stability of the robot. The maximum risk factor $\psi_{\max}$ refers to the highest level of risk encountered by a robot of a specific geometric configuration across all possible positions, considering all combinations of tilted substrate angle and joint angles, which is calculated with various the robot’s length $L_{1}$, and the length $L_{3}$ (between the gravity center and adhesion disk center) as $\begin{array}{c} \psi_{\text{max}}={\text{max}}_{\alpha_{0}\in \left[0,360\right],\alpha_{1}\in \left[0,100\right]}\left(\psi_{\left(\alpha_{0},\alpha_{1}\right)}\right) \end{array}$. The results in Fig. 4E show that when the length $L_{3}$ is fixed, the maximum risk factor $\psi_{\max}$ interestingly indicates an overlapping optimal point at $\psi_{\max}$ = 64%. Increasing $L_{1}$, equivalent to increasing $L_{2}$, supporting force distributes less on the right roller $B_{2}$ but more on wheels, improving stabilities. Further increasing $L_{1}$ over the kink point, the left roller $B_{3}$ becomes the main supporting point instead of the right roller $B_{2}$. The existing risk factor $\psi_{\max}$ implies optimal solutions of lever arms $L_{x}\left(x=1,\;2,\;3\right)$. The correlation is demonstrated as follows:

$$L_{1}+L_{2}={\mathrm{CL}}_{3}$$ (13)

where *C* is a scale factor. The *C* values are calculated as 1.2 with various $L_{1}$ and $L_{2}$, and fixed $L_{3}$. The correlation between the predefined geometric parameter $L_{3}$ and the scale factor was examined. When this geometric parameter was varied from 0 to 60 mm, as 60 mm is the maximum size for the robot, results show the C values are still around 1.2, and the error is less than 1% (shown as the inset in Fig. 4E). Hence, *C* is considered to be a constant, guiding robot structure design (like dimensions and wheel positions) and mass distribution center.

We attribute this optimized to the complex alternating force conditions on the 2 roller wheels during surface traversal, driven by gravitational moment directionality. This force alternation (evident as the discontinuity point in Fig. 4C) modulates the overall load distribution. The parameters $L_{1}+L_{2}$ and $L_{3}$ represent the gravitational lever arms for the respective roller wheels. When their lengths conform to the specific relationship, the system achieves a minimized maximum hazard coefficient, effectively preventing excessive loading on either roller wheel. Consequently, this critical constant arises intrinsically from the robot’s structural configuration, center of gravity distribution, and dynamic motion patterns.

According to the above stability analysis, we adjusted the mass center to meet the design criteria and concluded the dimensions in this work as $L_{1}=27.5\;\mathrm{mm},L_{2}=4.7\;\mathrm{mm},L_{3}=27.3\;\mathrm{mm}$.

### Locomotion characterization

**Fig. 5.**
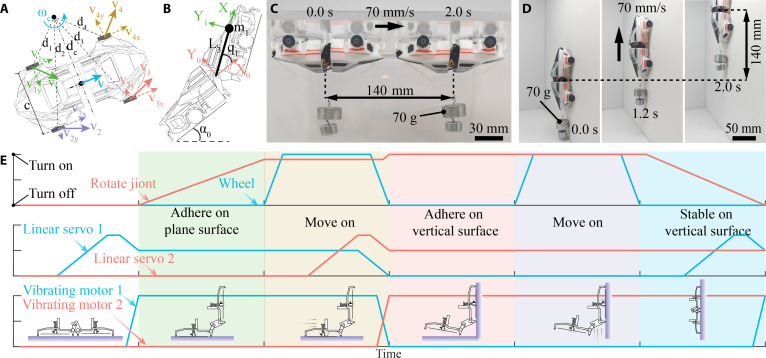
The motion analysis of the dual-unit wall-climbing robot. (A) Schematic of the dynamic model for the wall-climbing robot’s motion dynamic model. (B) Schematic of the dynamic model for the wall-climbing robot’s surface-traversing servo dynamic model. (C and D) Images of a robot climbing carrying a 70-g load on ceilings and vertical walls. (E) Sequence diagrams of cooperated joints, servos, and motors.

Dynamic models are fundamental for precise robotic motion control. Given the form of the robot in this paper, its dynamic modeling can be divided into 2 parts: the wheeled chassis motion model and the surface-traversing servo motion model, schematically illustrated in Fig. 5A and B. For the chassis kinematic model, given the consistent angular velocity across all points of the robot, we derive the following relationship:

$$\omega_{c}=\omega_{i}=\frac{v_{i}}{d_{i}}\left(i=1,\;2,\;3,\;4\right)$$ (14)

where *ω* denotes the rotational velocity and $d_{1\sim 4}$ represents the distance from the 4 wheels to the rotation center, respectively. Accounting for relative tire positions (wheelbase is *c*), the individual tire speeds $V_{L}$ (velocities of the 2 left wheels) and $V_{R}$ (2 right wheels) corresponding to the robot’s specific linear velocity $v_{x}$ and rotational velocity $\omega_{c}$ are obtained (detailed derivation provided in Text S5):

$$\left[\begin{array}{c} V_{L}\\ V_{R} \end{array}\right]=\left[\begin{array}{cc} 1 & -\frac{c}{2}\\ 1 & \frac{c}{2} \end{array}\right]\left[\begin{array}{c} v_{x}\\ \omega_{c} \end{array}\right]$$ (15)

For surface-traversing servo motion models (Fig. 5B), during cross-interface traversal, one end of the robot maintains continuous substrate contact, effectively serving as a fixed base; the opposing end undergoes servo-driven rotation. Applying the Lagrange method yields the dynamic equations governing this mechanical system (full derivation in Text S5):

$$\tau_{1}=m_{1}{L_{3}}^{2}{\ddot{q}}_{1}+m_{1}{\mathrm{gL}}_{3}\text{cos}\left(\alpha_{0}+q_{1}\right)$$ (16)

The surface-traversing servo force, denoted as $\tau_{1}$, is determined by several key parameters: the mass of half of the robot $m_{1}$, the length of the robot $L_{3}$, the tilted substrate angle $\alpha_{0}$, and the angle of the surface-traversing servo $q_{1}$. Integrating the established chassis motion dynamics with the surface-traversing servo motion model facilitates enhanced precision in controlling the robot’s holistic locomotion. This is achieved through dynamic adjustment of individual wheel and servo joint speeds, simultaneously improving mobility efficiency and adhesion stability. Crucially, the surface-traversing servo motion model reveals distinct torque components: the inertial torque (first term), which is generated by the system’s inertia during acceleration/deceleration, and the gravitational torque (second term), which is a configuration-dependent torque arising from gravity. This formulation is vital to prevent excessive velocities that could produce inertial torques exceeding the adhesive disk’s load capacity. Notably, during the robot’s slow, servo-actuated rotation, the inertial term becomes negligible. Omitting this term yields an equation consistent with the robot’s prior static analysis, confirming model coherence across dynamic and quasi-static regimes.

As a result of optimized acoustic adhesion and structure design, the robot can smoothly climb across obstacles and discontinuous surfaces, such as stairs and corners. The air layer beneath the disk creates minimal tangential resistance, resulting in motor-dominated propulsion dynamics. Notably, on vertical walls, the adhesion force manifested as normal pressure on the wheels, together with the driving motor torques, governs the loads. Considering the inverse speed–torque relationship inherent in compact motors, we selected a 30 gf·cm torque motor with 120 rpm capacity in this work (see Text S6) based on a systematic analysis of mobility and payload requirements. The climbing robot carried a 70-g payload (3.5 times its own weight) at a speed of 70 mm/s on ceilings and walls, as shown in Fig. 5C and D (for the full video, see Movie S3).

This absolute payload capacity of 70 g enables the deployment of diverse miniature detection devices within confined operational environments. Representative examples include high-definition endoscopic cameras (~20 g), miniature microphones (~30 g), or infrared probes (~60 g). In contrast, typical wall-climbing robots utilizing other adhesion methods engineered for higher absolute payloads typically possess substantially larger dimensions and mass, precluding access to narrow spaces.

When traversing concave right-angle corners, the robot elevates its front structure. This maneuver reduces the effective adhesion area and compromises stability due to gravitational torque, resulting in an approximate 30-g reduction in payload capacity compared to operations on flat surfaces. Furthermore, the complex traversing maneuvers required prevent the robot from achieving the traversal speeds feasible on unobstructed planar surfaces. Notably, traversing duration exhibits a strong correlation with operator proficiency. Consequently, automating the traversing process through motion trajectory planning utilizing LLMs can significantly reduce traversing time to 7 s, markedly enhancing traversal speed.

Automatic climbing involves multiple motors and servos, and we drew a diagram to explain these continuous steps of traversing a wall corner in Fig. 5E. The lines indicate individual motors or servos working on distinct steps. The robot features an integrated closed-loop feedback control system to ensure accurate execution of the movement strategies generated by the LLM. A detailed schematic of the control architecture is provided in Fig. S4.

Meanwhile, this robot is energy-efficient, with an average energy cost of 0.5 W for climbing or driving. For detailed specifications on the quality and power consumption of each component, please refer to Text S7.

**Fig. 6.**
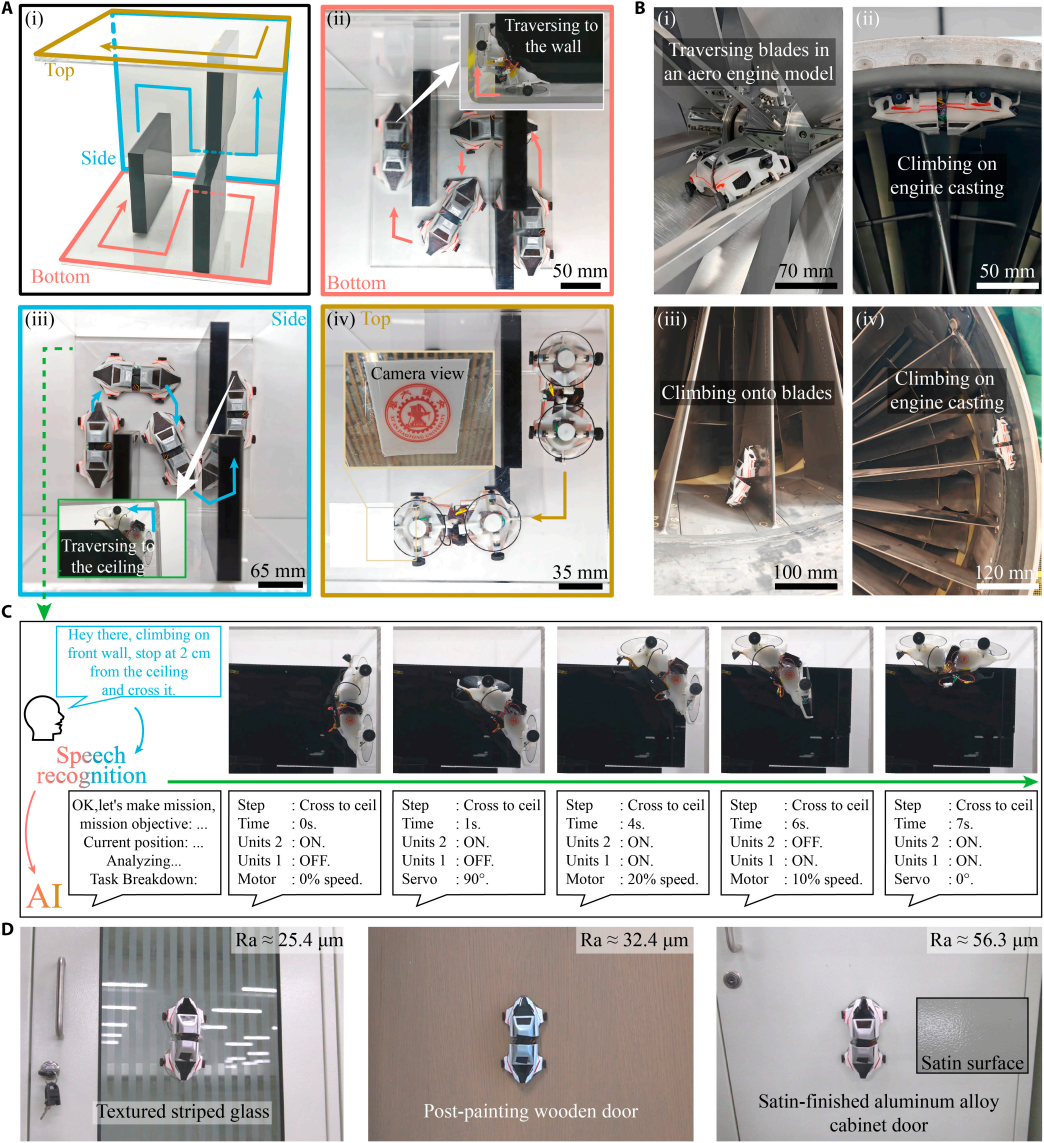
Demonstrations of target detection in a 3D maze and retired aircraft engines. (A) The climbing robot drove in a 3D maze (i), passed the narrow passage at its bottom (ii), traversed the corner to access the vertical walls (iii), and located the university logo on the ceiling (iv). (B) The robot navigated flexibly across blade surfaces (i) and inner walls (ii) of aircraft engine models, and successfully climbed on the curved blades (iii) and inside casings (iv) of retired aircraft engines using a flexible adhesion disk, demonstrating potential in rapid in situ damage detection in aero engines and other complex equipment cavities. (C) An example of AI-integrated motion control enables the robot to climb from a vertical wall onto an inverted ceiling. (D) Images demonstrate a wall-climbing robot traversing diverse nonsmooth surfaces. The robot exhibits robust surface adaptability, maintaining stable adhesion and locomotion on aluminum cabinetry, frosted glass, and painted wooden doors.

To illustrate the agility and intelligent detection capabilities of climbing robots, we made a complex 3D maze. The robot smoothly drove and navigated through the “N” curves, crossing corners to reach the ceiling, which was guided by AI (Fig. 6A and Movie S4). A picture of the university logo was finally identified and wirelessly recognized by a host computer, indicating potential applications in damage detection within space-confined machine cavities. By performing specialized modifications to cross-interface servos, the robot can also traverse convex right angles, as described in Text S8.

Moreover, the flexible anisotropic acoustic adhesive disk spontaneously conforms to curved surfaces, quantified specific curvature criteria of convex surfaces (radius ≥ 300 mm) and concave surfaces (radius ≥ 150 mm). Thus, the robot can climb on curved blades due to a flexible adhesion disk (Fig. 6B), potentially providing a novel and efficient strategy for in situ detection of aircraft engines and gas turbines. Additionally, the high temperatures in specific scenarios may negatively impact flexible disks. Inorganic soft materials, such as ceramics and glass, may replace polymeric disks for high-temperature applications [56,57].

Voice intelligence facilitates human–robot interaction by enabling robots to comprehend spoken language and thereby accept voice commands for simplified control. For instance, the robot—integrated with AI—was directed from a vertical wall to an inverted ceiling (Fig. 6C). Upon reaching the wall’s end (Fig. 6Aiii), it received the voice instruction “cross the ceiling corner 2 cm ahead”. A built-in microphone captured the speech and transmitted it to Google Cloud Speech-to-Text for conversion into text. This text was subsequently parsed by a pre-trained LLM (ChatGPT 4), which incorporates knowledge of the robot’s structure and functions, decomposing complex commands into discrete motion directives such as motor speeds, duration, adhesion unit activation, and joint angles (see Fig. S5 for LLM processing logic). As a result, the AI-integrated robot interprets voice commands and sequentially executes actions to traverse corners and transitions across vertical and inverted surfaces. Prior to operation, fundamental configuration data—including robot geometry, kinematics, and environmental parameters—are preloaded into the LLM. During execution, task-specific information such as scene dimensions, robot position, and detected targets are communicated via natural language commands without predefined syntax. These commands can be highly abstract, as the LLM autonomously infers their semantic intent. This approach markedly differs from conventional voice control systems, streamlining operation and reducing the technical skill required of users.

Since the anisotropic acoustic adhesion mechanism presented in this study relies primarily on atmospheric pressure for attachment, it imposes no specific requirements on the substrate material. Adhesion performance is influenced solely by surface roughness and topography. We demonstrated the robot’s climbing capability on diverse substrates representative of common nonsmooth surfaces encountered in daily environments (Fig. 6D), including frosted aluminum alloy cabinets (Ra = 56.3 μm), striped frosted glass (Ra = 25.4 μm), and painted wooden doors (Ra = 32.4 μm). This versatility highlights the robot’s robust adhesion across surfaces with complex texture.

## Discussion

To quantify and compare the climbing performance of different robots using various adhesion mechanisms, we introduce the concept of specific power intensity γ—power per unit mass and per unit characteristic length (dimension in the direction of movement) for wall-climbing robots, which effectively captures both adhesion strength (strong) and mobility flexibility (agile) in a unified framework.

$$\gamma =100g\frac{m_{\text{total}}}{m_{\text{self}}}•\frac{v}{L}=100\frac{P_{\mathrm{req}}}{m_{\text{self}}•L}\left[W•{\mathrm{kg}}^{-1}•m^{-1}\right]$$ (17)

where $m_{\text{total}}$ means the total mass including both payloads and robot’s weight $m_{\text{self}}$. The *v* and *L* note averaged driving speed and characteristic length (dimension in the direction of movement), respectively. *g* is gravity acceleration (9.8 m/s^2^). The coefficient 100 is used to convert the normalized speed *v*/*L* into a percentage.

**Fig. 7.**
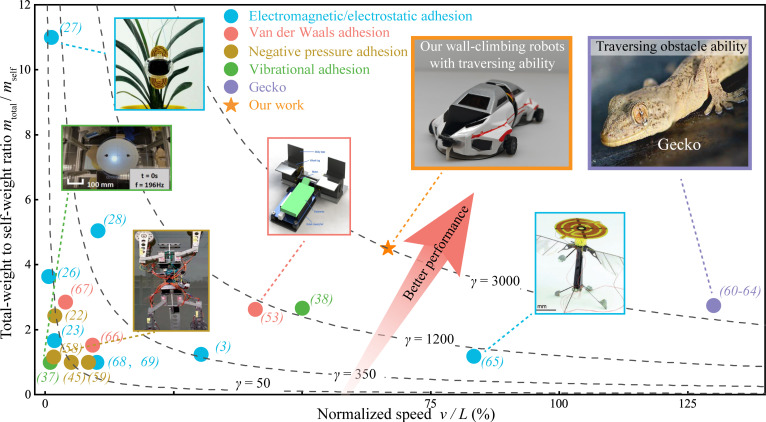
Performance comparisons of climbing robots considering both payloads and driving speeds on walls. According to the specific power intensity *γ* value, which represents the minimum power required to drive a loaded robot on walls, we compared and plotted the climbing performance of robots with various adhesion mechanisms. The horizontal axis corresponds to the velocity *v* of the wall-climbing robot normalized by its body length *L* along the direction of motion, expressed in body lengths per second. The vertical axis indicates the ratio of the total weight of the robot $m_{\text{total}}$, including payload, to its own weight $m_{\text{self}}$, which serves as a measure of the wall-climbing robot’s adhesion capacity. Our robot, utilizing anisotropic acoustic adhesion, demonstrates superior performance compared to other wall-climbing robots, comparable to that of a gecko.

The specific power intensity γ standardizes key performance parameters for wall-climbing robots, effectively isolating performance from variations in adhesion mechanisms, locomotion modes, and dimensional differences. This specific power intensity γ reflects the overall capability of wall-climbing robots in terms of both adhesion strength and climbing agile. Higher total-weight to self-weight ratio $m_{\text{total}}/m_{\text{self}}$ and increased normalized speed *v*/*L* each contribute to an enhanced specific power intensity. Based on it, a comprehensive comparison was carried out across various wall-climbing robots, with the corresponding results summarized in Table 1 and Fig. 7.

**Table 1.** Comparison table of various types of wall-climbing robots

Robot type	Adhesion principle	Total-weight to self-weight ratio ($m_{\text{total}}/m_{\text{self}}$)	Normalized speed (v/L)	Specific power density (*γ*)
Gecko [60–64]	Van der Waals dry adhesion	2.75	1.15	3,098.43
Our robot	Anisotropic acoustic adhesion	4.50	0.67	2,940.00
Anisotropic wheeled wall-climbing robot [38]	Vibration-based acoustic adsorption	2.67	0.50	1,306.67
Wheel-legged wall-climbing robot [53]	Van der Waals dry adhesion	2.63	0.41	1,053.07
Flapping-wing adhesive robot [65]	Electromagnetic/electrostatic adhesion	1.19	0.83	972.22
Quadrupedal crawling robot [28]	Electromagnetic/electrostatic adhesion	5.05	0.10	506.30
Quadrupedal crawling robot [3]	Electromagnetic/electrostatic adhesion	1.25	0.30	371.21
Wheel-legged wall-climbing robot [66]	Van der Waals dry adhesion	1.52	0.09	137.66
Bipedal inchworm robot [27]	Electromagnetic/electrostatic adhesion	11.00	0.01	127.47
Gecko-inspired quadrupedal robot [67]	Van der Waals dry adhesion	2.86	0.04	110.54
Three-legged alternating-motion robot [68]	Electromagnetic/electrostatic adhesion	1.01	0.10	98.49
Gecko-inspired quadrupedal robot [69]	Electromagnetic/electrostatic adhesion	1.00	0.10	98.02
Bipedal flipping and moving wall-climbing robot [59]	Vibration-based negative pressure adsorption	1.00	0.08	82.59
Multiple suction cup alternating track robot [45]	Negative pressure adhesion	1.00	0.05	49.75
Bipedal inchworm robot [22]	Negative pressure adhesion	2.43	0.02	44.20
Bipedal inchworm robot [23]	Electromagnetic/electrostatic adhesion	1.67	0.02	29.40
Quadrupedal crawling robot [26]	Electromagnetic/electrostatic adhesion	3.64	0.01	24.10
Gecko-inspired quadrupedal robot [58]	Vibration-based negative pressure adsorption	1.16	0.02	18.41
Centrifugal force-driven wall-climbing robot [37]	Anisotropic acoustic adhesion	1.00	0.01	9.80

According to performance comparisons in Fig. 7, Murphy et al. [66] proposed a medium-sized dry adhesion climbing robot with high speed. However, these types of robots had a small loading ratio due to limitations in volume and adhesion principles [53]. Gu et al. [27] designed a soft wall-climbing robot based on electrostatic adhesive. It could carry up to 11 times its weight, although its movement was relatively slow. In contrast, geckos hold their relatively heavy bodies and swiftly move on walls, indicating a high γ value of 3,098.

Targeting the geckos, the γ values of current reported climbing robots were compared and plotted in Fig. 7. It is noteworthy that existing vibration-based adhesion robots are also highlighted in the diagram. Gecko-inspired quadrupedal robot [58] and bipedal flipping and moving wall-climbing robot [59] employ vibration-driven negative pressure suction cups for adhesion, operating on a principle identical to that of conventional negative pressure suction cups—with the key distinction that a vibration motor replaces the vacuum pump to evacuate internal air. Unlike the anisotropic acoustic adhesion mechanism introduced in this work, this method demonstrates substantial tangential resistance. As a result, both systems are implemented in large-scale legged robots, which are unsuitable for inspection tasks in confined spaces, thereby limiting their practical application (with an average length of 50 cm). Another research group [37,38] has proposed a vibrating acoustic adhesion robot that generates adhesive force via the vibration of a 60-cm thin film.

Our study further refines the theoretical model for annular thin disk vibration and verifies its accuracy through repeated experiments. Innovatively, a 3D line laser scanner is employed to obtain precise measurements and monitor variations in the air gap thickness of the vibrating disk, thereby informing the design and control strategies to enhance adhesion strength.

Building on this theoretical framework, an active control system was developed to enable continuous adjustment of the acoustic adhesion force. This advancement addresses a key limitation of the reference robot, which relied on external pre-pressure for adhesion. Moreover, whereas the robot described in the reference literature was restricted to continuous surfaces, the robot presented here achieves stable and secure traversing across different surface through optimized structural design and mechanical modeling. This capability significantly broadens the operational scope of wall-climbing robots for diverse inspection scenarios.

Given the complex actuation and multi-degree-of-freedom nature of this wall-climbing robot, a motion planning and closed-loop feedback system based on an LLM was designed. This system supports voice-controlled execution of sophisticated tasks, fully leveraging the robot’s anisotropic acoustic adhesion and structural advantages. The climbing robots in this work utilizing optimal anisotropic acoustic adhesion demonstrate the best climbing performance taking into account both speeds and payloads, leading to a high γ value of about 2,900, approaching to that of a gecko. This work offers valuable insights for the development and deployment of compact yet high-performance wall-climbing robots, holding marked promise for widespread application in areas such as aircraft engine maintenance, gas turbine inspection, and a variety of other confined-space environments.

This robot demonstrates robust adhesion and mobility performance under controlled laboratory conditions and some real-world environments. Future enhancements to its performance are anticipated in 3 key areas: the adsorption mechanism, structural design, and control strategies. Achieving stable acoustic adhesion necessitates smooth surfaces to maintain a uniform, thin air layer. To improve adaptability for operation on rough or porous substrates—common in residential or disaster scenarios—future iterations could enhance the sealing integrity of the negative pressure cavity through structural modifications. Promising approaches include integrating variable-stiffness mechanisms and incorporating multi-scale surface features. Modular self-assembly presents a promising approach for reconfigurable robotics. Our untethered, on-board-electronics-equipped climbing unit serves as a potential building block for larger assemblies exhibiting collective behaviors. Combination strategies like magnetic coupling (Fig. S6) warrant investigation. The current prototype, fabricated via 3D printing using photosensitive resin, could benefit from material advancements; employing carbon fiber composites in future iterations offers potential for enhanced durability and reduced mass. Power is currently supplied by a lithium-ion battery with a rated 600-cycle lifespan, sufficient for operational requirements. Our AI-integrated control method requires manual error correction. In future research, embedding embodied intelligence capabilities could automate locomotion and obstacle navigation, overcoming this fundamental limitation for complex operational scenarios.

## Conclusion

In this paper, we demonstrate an AI-integrated climbing robot utilizing optimized anisotropic acoustic adhesion. We designed a wireless-controlled, 20-g intelligent robot that carries a 70-g payload and climbs both vertical and inverted surfaces at a speed of 70 mm/s. The robot navigates a 3D transparent maze and an aircraft engine. A single vibrating disk produces a partial vacuum that can lift 180 times its own weight (approximately 2 g). The disk diameter and vibration frequency are optimized to enhance adhesion by modeling the fluid mechanics. We designed a linear servo-spring system to regulate the critical air layer thickness (which determines the negative pressure) and adjust the adhesive force normal to the surface during the attaching and detaching processes. We analyzed and optimized the resonance between the vibrating disk and the robot body by selecting the appropriate spring stiffness and vibration frequency. To climb across noncontinuous surfaces, such as corners and stairs, we designed a rotating joint that combines 2 modular adhesion units. The climbing stability was further quantified, resulting in strict design criteria for the robot’s dimensions and center of gravity. The robot is equipped with a camera, demonstrating the potential for visual inspection of equipment in limited, narrow cavities. The camera can be augmented with other sensors to expand robot functionalities. The integration of AI enables climbing robots to understand voice commands, facilitating easy human–robot interaction and simple control. We evaluated a number of state-of-the-art wall-climbing robots with the proposed standardized specific power density intensity. The results indicate that the overall performance of our robot, in terms of specific power density, is comparable to that of a gecko. This study provides design criteria for high-performance, intelligent, wall-climbing robots suitable for a wide range of applications, including electronic manufacturing, machinery inspection, and disaster rescue.

## Materials and Methods

We set up a central controller for wireless communications based on ESP32S3 and designed an interrupt-driven program to guarantee real-time control (Fig. S7A). The entire system is powered by a 3.7-V, 100-mAh lithium battery.

We used a 3D line laser scanner (LNX-8030, Mech Mind, China) and a high-speed camera (T2410, Phantom, USA) to analyze the changes in the air layer. The adhesion force was measured using a universal tensile test machine (Wance TSE102A, China) with a stretching speed of 10 mm/min, as shown in Fig. S7B and C. The pressure within the negative pressure chamber of the vibration disk was monitored utilizing dynamic high-frequency pressure sensors (HIGHJOIN, China) and a 4-channel oscilloscope (Tektronix, USA). The laser scanner outputs a 2D array, indicating time-dependent profile contours. The laser can capture the vertical vibration of 4,096 points along the disk contour, relative to the stable substrate surface. Given the disk’s 50 mm diameter, this results in an approximate resolution of 12 μm. Following basic filtering and deflection correction, the processed data were plotted chronologically on a single graph (Fig. 2N). This visualization facilitates the systematic observation of temporal variations in the disk profile. The tensile testing machine features an integrated linear encoder and a strain gauge-based load cell, allowing for real-time recording of displacement and applied force during testing. The maximum force recorded in each test is designated as the ultimate tensile force of the adhesive disc under the specified parameters. A tensile text speed of 10 mm/min was employed to minimize inertial effects.

We used 3D printing (Formlabs Form 3, USA) to build the car frame and body. A vibration motor is glued onto the laser-cut flexible disk using a spin coater (Fig. S7D). The thin-film resistive pressure sensors (FSR IMS-C07A) were placed under wheels to quantify the wheel pressure generated by the vibration disk (Fig. S7E).

The linear servo features a 7-mm stroke and operates with control pulse widths of 1,500 to 2,100 μs, achieving ~20-μm positioning precision. Assembly errors and motor vibrations are absorbed by the spring, ensuring operational reliability. The control chip runs at 333 Hz; accounting for mechanical inertia and data processing latency, the response delay is ≤10 ms. This enables high-complexity fine-tuning and control capabilities. For details on communication latency and stability related to LLMs, see Text S9.

## Data Availability

All data needed to evaluate the conclusions in the paper are present in the paper and/or the Supplementary Materials. Additional data related to this paper may be requested from the authors.

## Supplementary Materials

Supplementary 1 Texts S1 to S9 Figs. S1 to S8 Table S1 Movies S1 to S4
