# Supplementary material for: Strong and Agile Wall-Climbing Robots Capable of Traversing Obstacles via Anisotropic Acoustic Adhesion
Source: Research (Wash D C). 2026 Jan 7;9:1038. doi: 10.34133/research.1038 (PMC12776589; doi:10.34133/research.1038)
Supplement: Supplementary 1 — Texts S1 to S9 Figs. S1 to S8 Table S1 Movies S1 to S4 [file research.1038.f1.zip › Supplementry Information.docx]

SUPPLEMENTARY MATERIALS

**Text S1. The action planning specification for large language models and the complete pre-training dialogues.**

Unlike conventional robots limited to 2D ground movement, wall-climbing robots traverse vertical or inverted surfaces using inherently 3D motion paths. This complex mobility, along with the risk of detachment due to operational errors, makes their path planning and motion control considerably more challenging than those of standard ground robots. Traditional control methods often depend on manual operation via wired controllers, requiring high operator expertise to perform real-time path planning based on environmental feedback while precisely coordinating multiple joint speeds and angles. The cross-interface wall-climbing robot studied here is designed for environments with complex geometries, such as aircraft engines. Compared to robots operating on large smooth surfaces, this robot must navigate right-angled interfaces, adding further difficulty. With up to nine controllable motors integrated internally, direct manual control of each joint is nearly infeasible.

Recent progress in artificial intelligence, especially large language models (LLMs), offers potential for breakthroughs in automated trajectory planning. However, current LLM applications are largely centered on humanoid robots, with little attention paid to specialized domains like wall-climbing robotics. This gap may arise from the non-humanoid morphology of such robots, which lacks the universal reference databases commonly used by LLMs for humanoid task planning.

To bridge this gap, we innovatively employed knowledge embedding to adapt GPT for motion planning in specialized wall-climbing robots. We first constructed a dedicated textual corpus covering robot specifications, dimensional parameters, basic motion patterns, and output formats. Using OpenAI’s Embeddings API, text segments were converted into embedding vectors. These custom knowledge units and vectors were stored in a vector database to enhance contextual comprehension. To balance response efficiency and accuracy, corpus indexing parameters were set as: maximum input tokens = 4,096, response tokens = 512, chunk overlap = 20, tokens per chunk = 600, and temperature = 0.7. When users submit task objectives via a standardized interface, GPT converts queries into vectors and performs semantic matching against both default and embedded knowledge. The refined output is then delivered via the chat API, as illustrated in **Fig. S5A**.

By embedding domain-specific knowledge—such as geometric parameters, preset motions, task examples, and output constraints—into the LLM, we enable it to generate professional motion and trajectory plans. We developed and validated a novel control framework combining "speech recognition + LLM motion planning + manual fine-tuning." Operators use natural language commands (e.g., “Find the school emblem”) captured by onboard microphones, transcribed into text, and processed by our pretrained LLM. The model then autonomously generates compliant motion trajectories as temporal sequences of joint states, which the robot executes directly. This approach eliminates the need for predefined syntax and abstracts complex operational intent, distinguishing it fundamentally from conventional voice control systems and greatly lowering the technical barrier.

Additionally, recognizing that robots in real-world settings often deviate from planned trajectories (e.g., due to dust or surface irregularities) and considering the lack of feedback sensors for closed-loop control, we incorporated a manual fine-tuning mechanism (**Fig. 1D**). When significant deviation occurs, the operator can intervene, pausing LLM-generated execution until manual corrections are applied.

Because both the overall strategy and execution steps are LLM-generated, operators only need to issue directional or minor corrective commands (e.g., “turn left” or “back up slightly”). This hybrid scheme thus reduces the difficulty and cognitive load of manual global planning while retaining essential human oversight and error correction.

Through this framework, we demonstrate the effectiveness and promise of LLMs in controlling non-humanoid specialized robots, as exemplified by our wall-climbing platform. Our approach reduces control complexity and operator burden while improving mobility efficiency. The core methodology—domain-knowledge embedding, natural language interaction, and strategic human oversight—holds strong potential for transfer to other specialized robotic systems, such as submersible or snake-like robots.

The text with knowledge embedded is as follows:

*Pre-training procedures*

1. Objectives:

I'll tell you some of the purposes of the robot, and you break down those purposes into tasks (since the tasks may contain some complex subtasks, I'll tell you exactly what you should do within the subtasks below), breaking them down into what the robot's joints need to do at each time. Your answer in English

1. Robot parameters:

The robot has four wheels: left front wheel (LF), right front wheel (RF), left rear wheel (LB), and right rear wheel (RB). They are at the four vertices of the rectangle (length and width are 52 mm, 53 mm)

In the middle of the robot (104 mm ×57 mm ×27 mm) there is a servo, which is able to lift the front part of the robot at an angle (alpha) from 0-90°.

The robot has two adhesion units (adhesion unit 1 and adhesion unit 2), which allow the front or back half of the robot to be sucked onto the floor or a wall. Each adhesion unit has a linear servo to control the distance, named respectively: liner servo 1; liner servo 2. The two linear servos have a range of -10 to 10, with a zero-value indicating that the adhesion disk contacts the substrates. The negative means “drop”, a positive means “pull up”.

1. Robot Motion:

The robot moves and steers on four wheels, like a tank.

4. Robot Subtask - Step Crossing Procedure. 1:

4.1. pull up the “rear linear servo (liner servo 2)”, activate the “rear adhesion unit (adhesion unit 2)”, control the “servo” to move to 90°, control the “servo” to move to 90°, and control the “servo” to move to 90°. Move the “servo” to 90° and lift the “front part”. 2.

4.2. Move forward, when the lifted “front half” reaches the top of the step, control the “Servo” to return to 0°, lower the “front half”, and turn off the “rear adsorption unit (Servo)”. “Turn off the rear adhesion unit (adhesion unit 2), lower the rear linear servo (linear servo 2), lower the front linear servo (linear servo 1), start the rear linear servo (linear servo 2), and turn on the front linear servo (linear servo 1). Lower the “rear linear servo (liner servo 2)”, lower the “front linear servo (liner servo 1)”, and start the “front adhesion unit (adhesion unit 1)”. 3.

4.3. Move the servo to 90° again, and lift the rear part of the servo as the front part is held. 4.

4.4. Pull up the “Front linear servo (Liner servo 1)” and keep the “Front adhesion unit (adhesion unit 1)” on. Advance until the back half is above the steps, control the servo back to 0° and lower the back half.

5. Robot Subtask - Cross Interface Process:

5.1. Pull up the rear linear servo, activate the rear adhesion unit, control the servo to move to 90°, control the servo to move to 0°, and lower the rear half. Move the “servo” to 90° and lift the “front part”. 2.

5.2. Move forward, when the lifted “front part” is attached to the wall, turn off the “rear adhesion unit (adhesion unit 2)”, lower the “front linear servo (liner servo 1)”, and start the “front linear servo (servo 2)”. Turn off the “rear adhesion unit (adhesion unit 2)”, lower the “front linear servo (liner servo 1)”, and start the “front adhesion unit (adhesion unit 1).

5.3. Forward, because at this time the “front part” has been adsorbed on the wall, so the robot will climb the wall and go upward, wait until the “back part” leaves the ground for a certain distance, control the “servo” to return to 0 °, turn the “servo” back to 0 °, turn the “servo” back to 0 °, turn the “servo” back to 0 °. After the “rear part” has left the ground for a certain distance, control the “Servo” to return to 0°, put the “rear part” onto the wall, and then activate the “adhesion unit 2”.

6. Example of return format:

time: 0.1s

LF-forward-70%speed

RF-forward-70%speed

LB-forward-70%speed

RB-forward-70%speed

Servo-23°

adhesion unit1-ON

adhesion unit2-OFF

liner servo 1:5

liner servo 2: 7

time: 0.5s

LF-forward-70%speed

RF-backward-50%speed

LB-forward-70%speed

RB-backward-50%speed

Servo-56°

adhesion unit1-OFF

adhesion unit2-OFF

liner servo 1:3

liner servo 2: -2

7. Example of use:

I ask: There is a step 5cm in front of the robot, after the step, turn left 3cm and move to a wall interface.

What you should think about: first “go forward”, then break down the task as shown in “Robot Subtask - Cross Step Process”, then “go forward and turn left” again, then move to the corner of the wall as shown in “Robot Subtask - Cross Step Process”, then “go forward and turn left” again. “Robot Subtasks - Crossing Interfaces”, then ‘Forward’ to reach the wall.

8. Your answer:

time: 0.1s

LF-forward-100%speed

RF-forward-100%speed

LB-forward-100%speed

RB-forward-100%speed

Servo-0°

adhesion unit1-OFF

adhesion unit2-OFF

liner servo 1: 0

liner servo 2: 0

(Move to the front of the step)

time: 0.5s

LF-forward-50%speed

RF-forward-50%speed

LB-forward-50%speed

RB-forward-50%speed

Servo-90°

adhesion unit1-OFF

adhesion unit2-ON

liner servo 1: 0

liner servo 2: 5

...... (This sentence belongs to the comment category and is not something you should answer. The ellipses here indicate specific answers within the “Robot Subtask - Step Crossing Process”)

(The robot has crossed the step.)

time: 6s

LF-backward-50%speed

RF-forward-50%speed

LB-backward-50%speed

RB-forward-50%speed

Servo-0°

adhesion unit1-OFF

adhesion unit2-OFF

liner servo 1: 0

liner servo 2: 0

(Robot reaches the front of the corner)

time: 7s

LF-forward-30%speed

RF-forward-30%speed

LB-forward-30%speed

RB-forward-30%speed

Servo-90°

adhesion unit1-OFF

adhesion unit2-ON

liner servo 1:0

liner servo 2: 5

...... (This sentence belongs to the comment category and is not something you should answer. The ellipsis here indicates a specific answer within the “Robot Subtask - Cross-Interface Process”)

(The robot has gone over the wall.)

time: 12s

LF-forward-100%speed

RF-forward-100%speed

LB-forward-100%speed

RB-forward-100%speed

Servo-0°

adhesion unit1-ON

adhesion unit2-ON

linear servo 1:5

liner servo 2: 5

(Robot reaches the target position and stays)

time: 13s

LF-forward-0%speed

RF-forward-0%speed

LB-forward-0%speed

RB-forward-0%speed

Servo-0°

adhesion unit1-ON

adhesion unit2-ON

liner servo 1:5

liner servo 2: 5

9. Robots should be prioritized when completing tasks:

Logic, does it make sense in the physical world

10. Communication or tone of voice:

Think of it as feedback given by the computer, no need to be too emotional

*Complete response with AI (Take the action in the* ***Fig. 1C*** *as an example)*

Step 1: Move

LF, RF, LB, RB: Forward motion at 100% speed for 8 cm.

Adhesion Units: OFF.

Step 2: Anchor to the surface

LF, RF, LB, RB: Forward motion at 0% speed.

Liner Servo 2: -2.

Adhesion Unit 2: Activate to adhere to the surface.

Liner Servo 2: 5.

Servo: Adjust angle to 90° (preparing to lift).

Step 3: Move

LF, RF, LB, RB: Forward motion at 50% speed for 5 cm.

Step 4: Cross stair

LF, RF, LB, RB: Forward motion at 0% speed

Adhesion Units: OFF.

Servo: Adjust angle to 45°.

Liner Servo 2: -5.

Servo: Adjust angle to 0°.

Liner Servo 2: -10.

Liner Servo 1: -2.

Adhesion Unit 1: Activate to adhere to the surface.

Liner Servo 1: 5.

Servo: Adjust angle to 90°.

LF, RF, LB, RB: Forward motion at 50% speed

Servo: Adjust angle to 0°.

LF, RF, LB, RB: Forward motion at 0% speed

Step 5: Contact the wall

Liner Servo 2: -2.

Adhesion Unit 2: Activate to adhere to the surface.

Liner Servo 2: 5.

LF, RF, LB, RB: Forward motion at 0% speed

Adhesion Units 1: OFF.

Servo: Adjust angle to 90°.

LF, RF, LB, RB: Forward motion at 30% speed

LF, LB: Forward motion at 30% speed

RF, RB: Backward motion at 30% speed

LF, RF, LB, RB: Forward motion at 10% speed

LF, RF, LB, RB: Forward motion at 0% speed

Liner Servo 1: -2.

Step 6: Climb cross to wall

Liner Servo 1: -2.

Adhesion Units 1: ON.

Liner Servo 1: 5.

LF, RF, LB, RB: Forward motion at 0% speed

Adhesion Units 2: OFF.

Servo: Adjust angle to 90°.

LF, RF, LB, RB: Forward motion at 30% speed

Servo: Adjust angle to 0°.

LF, RF, LB, RB: Forward motion at 0% speed

Step 7: Contact the ceil

Adhesion Units 2: ON.

LF, RF, LB, RB: Forward motion at 0% speed

Adhesion Units 1: OFF.

Servo: Adjust angle to 45°.

LF, RF, LB, RB: Forward motion at 30% speed

Servo: Adjust angle to 90°.

LF, RF, LB, RB: Forward motion at 10% speed

Liner Servo 1: -2.

Adhesion Units 1: ON.

Liner Servo 1: 5.

Step 8: Climb cross to ceil

LF, RF, LB, RB: Forward motion at 0% speed

Adhesion Units 2: OFF.

Adhesion Units 1: ON.

Servo: Adjust angle to 90°.

LF, RF, LB, RB: Forward motion at 30% speed

LF, RF, LB, RB: Forward motion at 0% speed

Servo: Adjust angle to 0°.

Liner Servo 2: -2.

Adhesion Units 2: ON.

Liner Servo 2: 5.

Camera: Image Acquisition

The standardized embedded corpus file is as follows:

[

{

"metadata": {

"robot_id": "wall_climber_v1",

"task_type": "step_crossing",

"created_at": "2025-09-28T14:59:00Z"

},

"parameters": {

"chassis": { "length_mm": 104, "width_mm": 57, "height_mm": 27 },

"wheelbase": { "longitudinal_mm": 52, "lateral_mm": 53 },

"servo": { "angle_range": [0, 90], "unit": "degrees" },

"adhesion_units": [

{

"name": "unit_1",

"linear_servo": { "range": [-10, 10], "zero_position": "contact" }

},

{

"name": "unit_2",

"linear_servo": { "range": [-10, 10], "zero_position": "contact" }

}

]

},

"control_sequence": [

{

"time_s": 0.0,

"wheels": {

"LF": {"action": "forward", "power": 1.0},

"RF": {"action": "forward", "power": 1.0},

"LB": {"action": "forward", "power": 1.0},

"RB": {"action": "forward", "power": 1.0}

},

"servo_angle": 0,

"adhesion": {"unit_1": false, "unit_2": false},

"linear_servos": {"servo_1": 0, "servo_2": 0},

"annotation": "Approaching step"

},

{

"time_s": 2.1,

"wheels": {"all": {"action": "forward", "power": 0.5}},

"servo_angle": 90,

"adhesion": {"unit_1": false, "unit_2": true},

"linear_servos": {"servo_1": 0, "servo_2": 5},

"annotation": "Lift front, anchor rear"

},

{

"time_s": 3.8,

"wheels": {"all": "stop"},

"servo_angle": 0,

"adhesion": {"unit_1": true, "unit_2": false},

"linear_servos": {"servo_1": 5, "servo_2": -10},

"annotation": "Lower front, engage adhesion"

},

{

"time_s": 4.2,

"servo_angle": 90,

"linear_servos": {"servo_1": 5, "servo_2": -5},

"annotation": "Lift rear section"

}

]

},

{

"task_type": "wall_transition",

"control_sequence": [

{

"time_s": 0.0,

"adhesion": {"unit_1": false, "unit_2": true},

"servo_angle": 90,

"linear_servos": {"servo_2": 5},

"annotation": "Anchor to ground, lift front"

},

{

"time_s": 1.7,

"wheels": {"all": {"action": "forward", "power": 0.3}},

"trigger_condition": "front_sensor.contact_wall == true"

},

{

"time_s": 2.4,

"adhesion": {"unit_1": true, "unit_2": false},

"linear_servos": {"servo_1": 5, "servo_2": -3},

"annotation": "Switch adhesion to wall"

},

{

"time_s": 3.1,

"servo_angle": 0,

"wheels": {"LF": 0.3, "RF": 0.3, "LB": 0.3, "RB": 0.3},

"trigger_condition": "pitch_sensor > 75"

}

]

}

]

**Text S2. The derivation process of the anisotropic acoustic adhesion theory.**

The relationship between the adhesive force and the dimensionless force in anisotropic acoustic adhesion is as follows:

The expressions for the Stokes number and the acoustic wave number are as follows:

where is the aerodynamic viscosity (0.018 mPa·s, 20℃) and is the air density (1.2 kg/m3). The and can be expressed as:

The represents the correction factor used to adjust for deviations caused by environmental changes and gas non-uniformity. To expand the above expression, we first define two auxiliary transverse functions [1]:

Then , and in the above equation can be expressed as:

The denotes the integral function along the radius:

where the auxiliary function is:

Where and are *n*-order Bessel functions of the first kind and second kind, respectively. The is the normalized radius.

**Text S3. Detailed calculation procedure for resonance analysis.**

The force balance equation is proposed as:

where ,, and are the quality matrix, damping matrix, stiffness matrix and displacement matrix respectively as:

The is the total external force, includes adhesion force as:

where notes an average external force under the combined action of vibrational and adhesion, . The near-field acoustic adhesion’s amplitude is deduced by solving above equations, which is a differential equation with a generalized solution of:

Bringing in the solution gives:

Its dimensionless expression is:

In this formula, the symbol denotes the static deformation of the disk (corresponds to the external force at a frequency of zero); represents the mass ratio; indicates the ratio of natural frequencies; corresponds to the damping ratio; refers to the excitation frequency ratio; signifies the natural frequencies of the robot; and stands for the natural frequencies of the disk.

**Text S4. Detailed calculation process for robot stability.**

The complete robot structure is schematically shown in **Fig. S3**. We use coordinate system transfer method to move the force received by each part to rear part 1, and take the axes of each part, the three coordinates of each gravity center are:

The ordinal numbers in the upper-right corner indicate various coordinate system for this location. We use transfer matrix to unify them into the original coordinate system:

where the coordinate transformation matrix is given by:

The torques of part 2 and part 3, for the original coordinate, are obtained obviously:

where . The state of force equilibrium of part 1 at this point is:

where , and mean the contact force from wheel, roller and roller respectively. These are dimension parameters. The plus or minus of resultant moment determines the working state of two rollers. Specifically, the right roller provides support moment and the left roller slightly suspends when , and vice versa when .

Due to the great normal adhesion performance mentioned above, the robot is quite stable when flat on the ground or hanging on the ceiling. However, as the robot climbs on the wall, friction provides all the force to resist gravity. Therefore, the limitation depends on friction instead of adhesion in this state, the max friction on wheel is defined as follows:

**Text S5. Derivation Process of the wheeled chassis motion model and the surface-traversing servo motion model**

The dynamic model of this robot can be divided into two parts: the wheeled chassis motion model and the surface-traversing servo motion model.

The kinematic model of the robot chassis, illustrated in **Fig. 5A**, was established to characterize its dynamic behavior. Based on the principle of consistent angular velocity relative to the instantaneous center of rotation (ICR) across all configurations of the robot, the following relationship is derived:

This fundamental angular velocity consistency allows the formulation to be generalized as:

Defining the angle between the line connecting each wheel to the ICR and the y-axis as , the geometric configuration yields:

To optimize locomotion efficiency, wheels on the same lateral side of the robot are constrained to rotate at identical speeds, resulting in:

combining above Equations provides the comprehensive relationship:

Leveraging this chassis dynamic model, the requisite rotational speeds for each drive motor can be computed to achieve a specified target translational velocity and rotational angular velocity, thereby enhancing the maneuverability performance of the wall-climbing robot.

For the surface-traversing servo motion model (**Fig. 5B**), although the robot's motion is relatively simple, we employed the LaGrange method to establish its servo dynamics model. This approach anticipates scalability for subsequent multi-joint robotic systems (**Fig. S6**). The LaGrange dynamics equations are formulated as follows:

where , is the kinetic energy and is the potential energy. To derive the system's kinetic and potential energies, we assume that half of the robot’s body remains rigidly fixed to the base at all times. This stationary half-body segment serves as the base reference frame, with its coordinate axes aligned with the servo axes. The other half-body segment with mass of is elevated at an angle of , with its coordinate system centered at the center of gravity and positioned at a distance . This gravity center configuration aligns with the static model dimensions detailed earlier. The kinetic energy of the robotic section (Section 2) is derived as follows:

The corresponding potential energy is given by:

Thus, the dynamic equation is computed as follows:

**Text S6. Drive motor analysis.**

The robotic system with total system mass adheres to a vertical surface governed by the force equilibrium:

where , , and respectively denote adhesion force, friction force, and friction coefficient. The locomotion velocity and motor torque exhibit the relationship as:

with representing wheel radius and corresponding to rotational speed (rpm). Under maximum power conditions, the motor's torque-speed characteristics simplify to , which when combined with above yields the fundamental constraint:

This parametric relationship establishes an inherent trade-off between mobility and payload capacity. Through evaluation of micromotor specifications, we chose a 30 gf**·**cm torque hollow-core motor operating at 120 rpm, optimally balancing dynamic performance and mechanical property within spatial constraints.

**Text S7. Mass and Power Consumption of Wall-Climbing Robot Internal Components**

The mass and power consumption of the robot's internal components are detailed in **Table S1**. The total mass for each component type is calculated as the product of its individual mass and quantity. Power consumption refers to the aggregate rated power draw for all identical units of a given component. To accurately reflect the intermittent operation of these components during a single surface-traversing movement cycle, the average power consumption was determined by multiplying each component's rated power by its corresponding operational duty cycle (i.e., the proportion of time it is active during the cycle).

**Text S8. Schematic diagram of a wall-climbing robot traversing a convex right angle.**

To facilitate obstacle traversal, the robot employs a surface-traversing servo motor offering a 250° range of motion, theoretically enabling the negotiation of convex right-angle obstacles. However, to minimize overall size and prevent servo damage, the motor is mounted at an offset angle as **Fig. S8**. In this configuration, the position where the front and rear sections align side-by-side defines one rotational endpoint (allowing 250° clockwise rotation from this position). For enhanced convex and concave right-angle obstacle clearance, the servo mounting angle can be optimized as depicted in **Fig. S8**. Here, the aligned position is defined as the center point, permitting rotation from +125° (clockwise) to -125° (counterclockwise). While this alternative configuration broadens obstacle-clearing capabilities, it increases the robot's footprint, particularly its length. Consequently, the flexibility and maneuverability in typical operational scenarios are inferior to the compact design presented in this work.

**Text S9. The interaction latency and recognition rate between speech recognition and large language models.**

The performance of AI-based voice interaction systems is highly dependent on network conditions. Laboratory experiments were conducted to evaluate latency under controlled network parameters. When utilizing the Xi'an Mobile Education Network (upload: 13.5 Mbps, download: 52.4 Mbps, mean jitter: 50 ms), connection latency to Google Speech Recognition + ChatGPT measured 156 ms (speech processing) + 282 ms (LLM response). In contrast, Baidu Speech Recognition + Deepseek required 89 ms + 132 ms. For end-to-end voice-to-dialogue processing via API interfaces, average latencies were 583 ms and 268 ms respectively.

Background noise and environmental interference reduced speech recognition accuracy to approximately 89%. However, the robust semantic parsing capabilities of large language models effectively compensated for minor recognition errors, ensuring these inaccuracies did not impact final decision-making outcomes.

**Table S1. The weight and power consumption of the internal components of the wall-climbing robot.**

| **Parts** | **Brand** | **Mass (g)** | **Rated power (W)** | **Percentage of time traversing interfaces (%)** | **Average power (W)** |
| --- | --- | --- | --- | --- | --- |
| Seeed Studio XIAO ESP32S3 Sense (Light Sleep Mode) | Seeed Technology (China) | 1.3*1=1.3 | 0.0185 | 80 | 0.0148 |
| 4-13 Hollow Cup Vibration Motor | Nidec (Japan) | 0.9*2=1.8 | 0.444 | 60 | 0.2664 |
| AGF-rc c1.5CLS 1.5g linear servo | AGFRC (China) | 1.1*2=2.2 | 0.148 | 15 | 0.0222 |
| DS-power DS-M005 servo | DS-power (China) | 1.2*2=2.4 | 0.185 | 15 | 0.02775 |
| 6mm Hollow Cup Planetary Gear Motor | KLSY (China) | 1.0*4=4.0 | 0.15 | 60 | 0.09 |
| Battery (3.7V 50mAh) | KeXiangWei (China) | 0.9*1=0.9 | N/A | N/A | N/A |
| Body Main Structure (Hollow photosensitive resin) | N/A(Not Applicable) | 1.5*2=3.0 | N/A | N/A | N/A |
| Cables | N/A | 0.5*1=0.5 | N/A | N/A | N/A |
| **Total** |  | 20.1 |  |  | 0.51 |


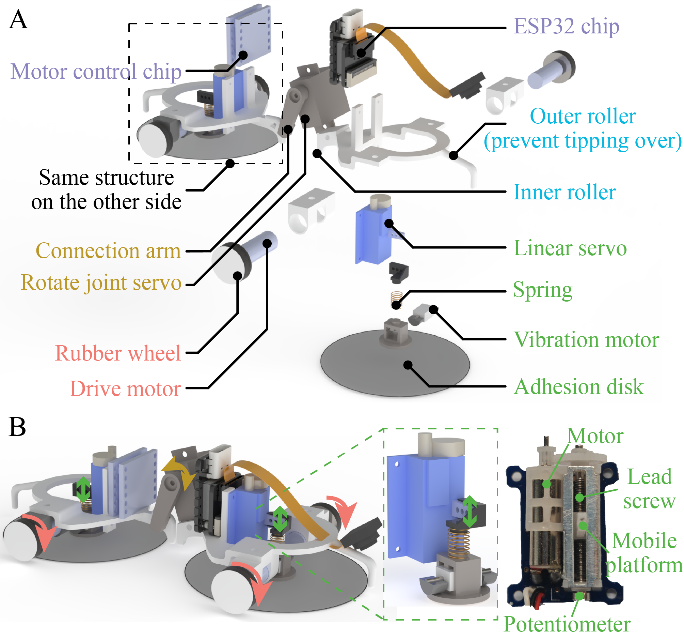


**Fig. S1.Detailed structural diagram of the robot.** (A) Exploded view of the robot's internal architecture. Five core components are color-coded: adhesion/active control unit (green), motor-integrated drive wheels (red), primary structural frame with inner/outer roller wheels (blue), surface-traversing servo mechanism with connecting arms (brown), and central control chip (purple). (B) Schematic of robotic motion joints and linear servo assembly. This servo integrates a motor, potentiometer, lead screw, and moving platform. Upon receiving control signals, its embedded driver circuitry modulates motor rotation, which is translated into precise vertical displacement of the platform via the lead screw. The integrated potentiometer provides real-time linear displacement measurement, enabling closed-loop control through instantaneous error correction.


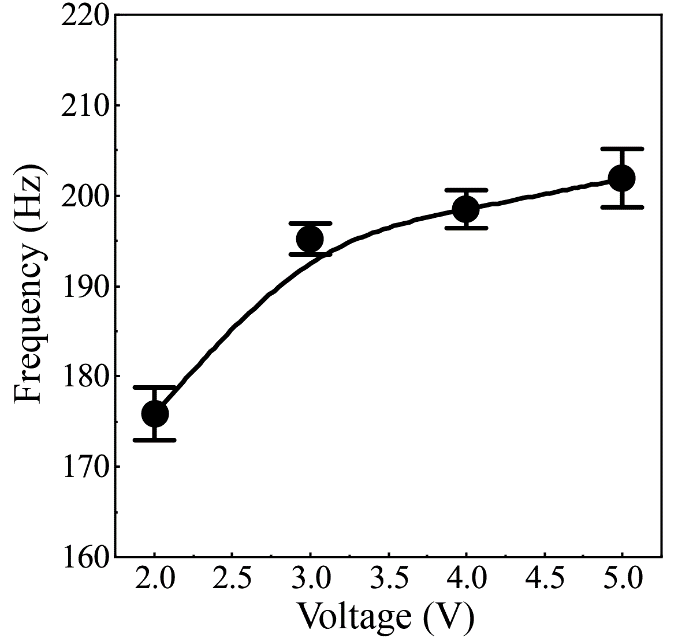


**Fig. S2. Voltage-frequency relationship of 0.8g vibration motors.** The voltage-frequency characteristics of the vibration motors were quantified using a laser tachometer (DLX-ZSY2301). Rotational frequency measurements were recorded across the operational voltage range (2-5 V, determined by motor rated capacity) with five replicate measurements at each voltage level.


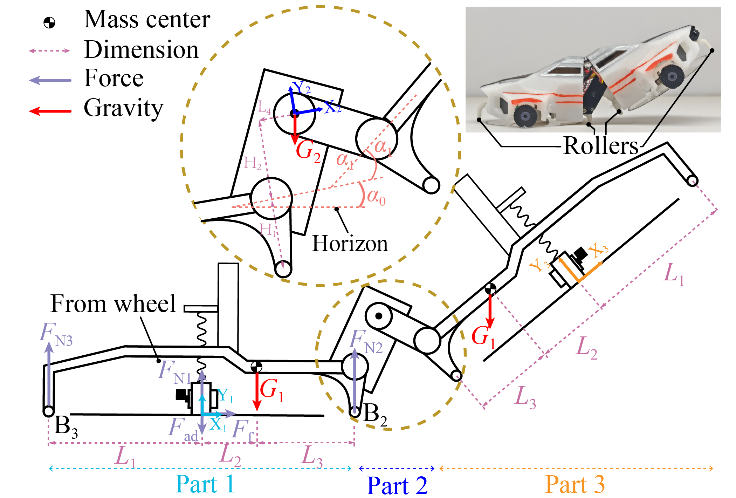


**Fig. S3. Diagram of robot structure details.**


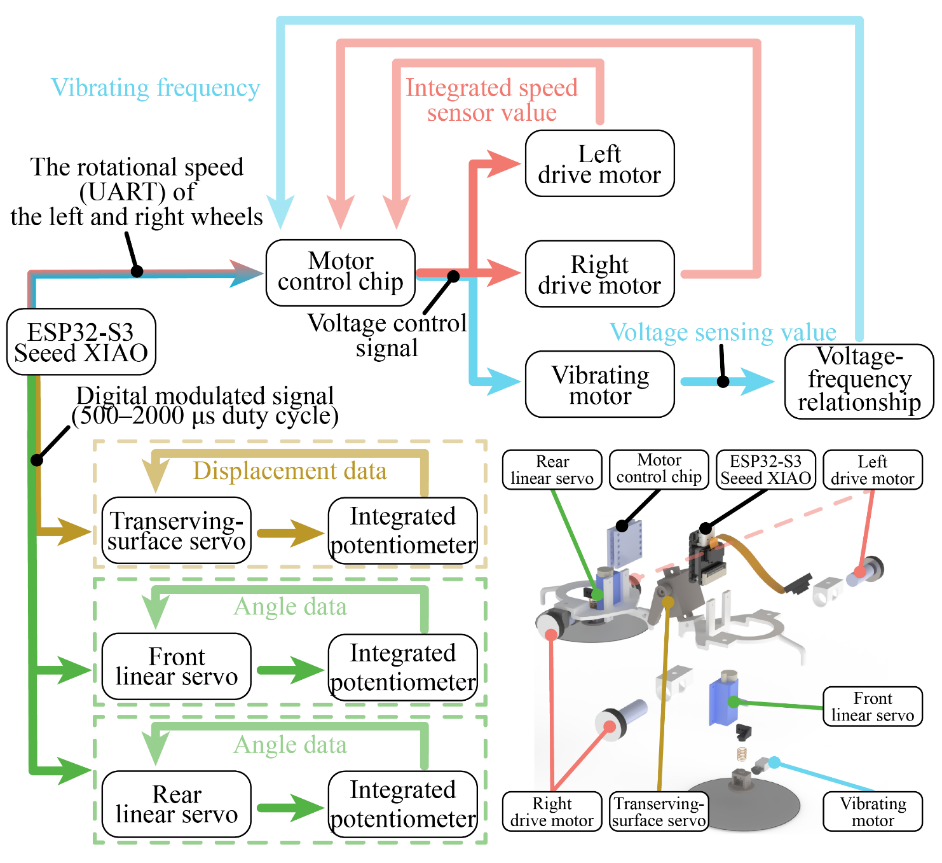


**Fig. S4**. **The block diagram of the wall-climbing robot control system.** The internal drive components achieve closed-loop motion control through integrated feedback mechanisms. Owing to the small form factor of the vibration motors, it is not feasible to mount speed sensors directly. Instead, the vibration frequency is derived from acquired voltage data, based on the voltage-to-frequency conversion relationship (**Fig. S2**) outlined in the text. Furthermore, the dashed-line enclosures around the three servos indicate that the feedback mechanism is embedded within the chips inside these servo units.


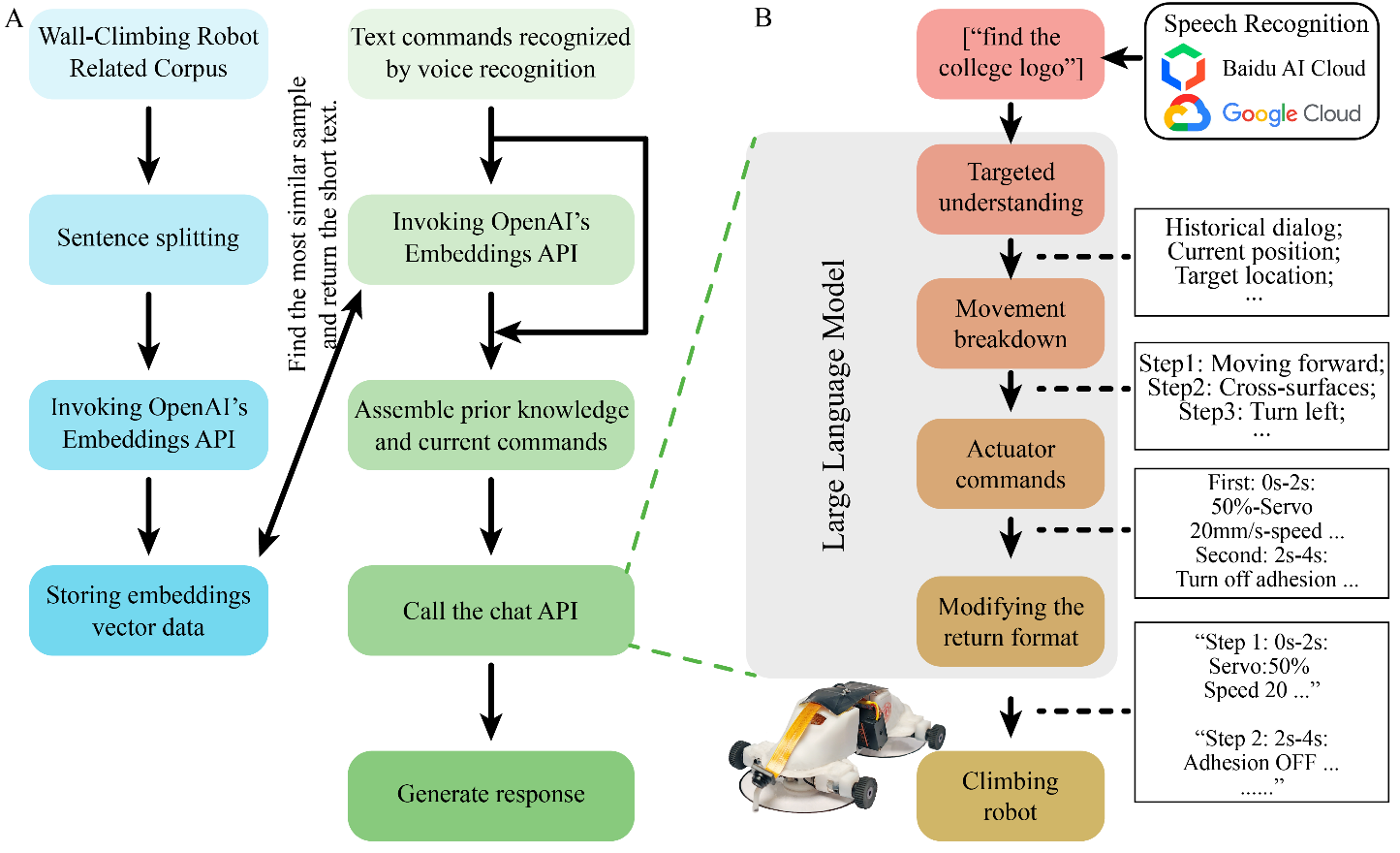


**Fig. S5. Large language models facilitate the task decomposition process.** (A) The knowledge embedding technology flowchart. By integrating this technology, we have achieved motion planning for specialized wall-climbing robots controlled by large language models. (B)The robot receives imprecise command text from the cloud for speech recognition and transmits it to the online large language model (ChatGPT-4, OpenAI, United States). The online large language model undergoes four steps to transform imprecise commands into a prescribed command format for execution by the robot.


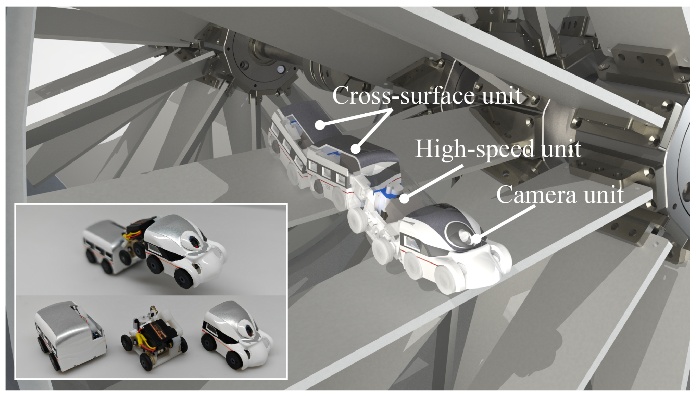


**Fig. S6.** **Schematic of self-assembling modular robots based on magnetic connection.**


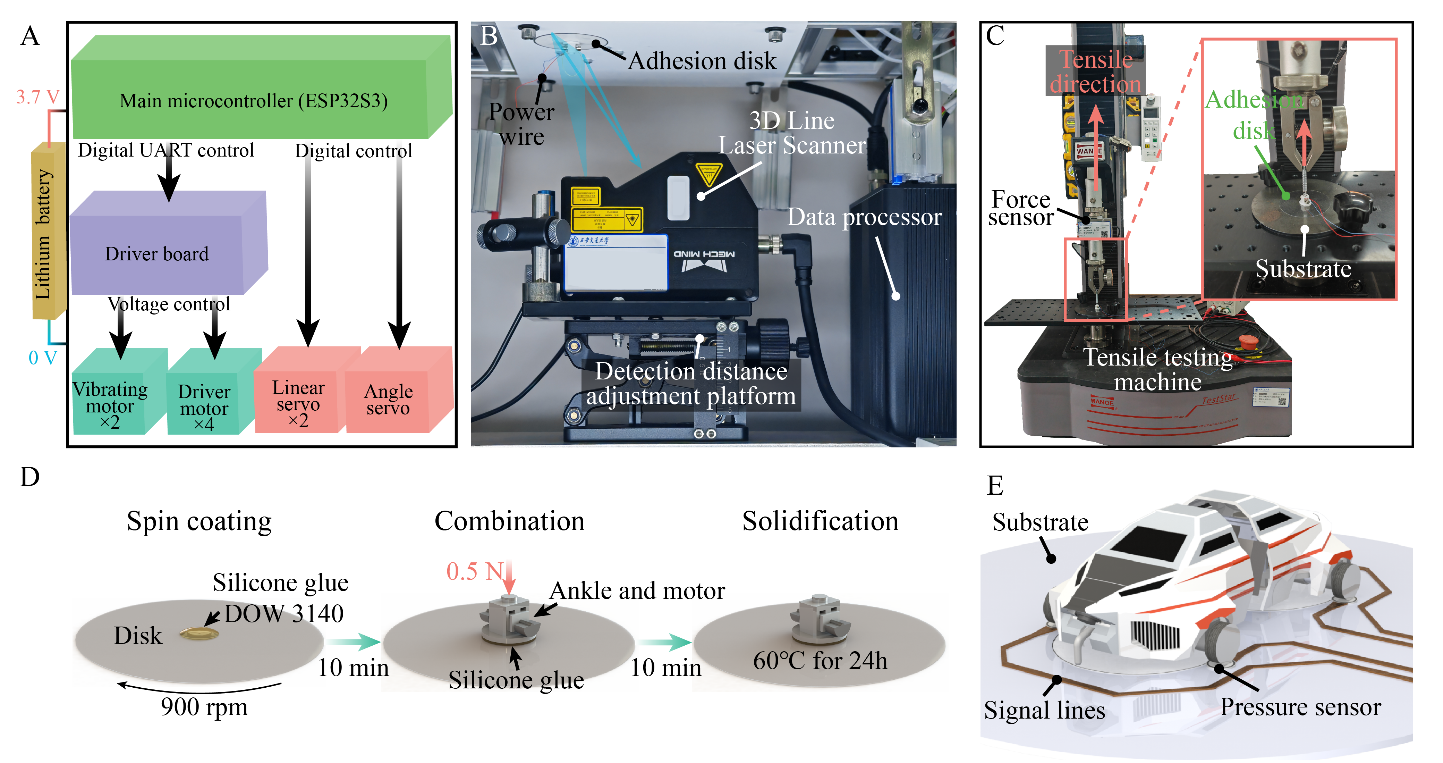


**Fig. S7.** **Control Logic Diagram and Experimental Methods.** (A) Control diagram of the robot. (B) The experimental diagram of 3D line laser scanning. (C) Imagine adhesion measurement using electro-mechanical universal testing machines. We picked copper as substrate and measured the force of disk away from substrate. (D) The fabrication process of vibrational adhesion disk. (E) Measurement method of wheel pressure. Thin-film pressure sensors are glued on the substrate, the robot adheres the substrate, and presses the sensors.


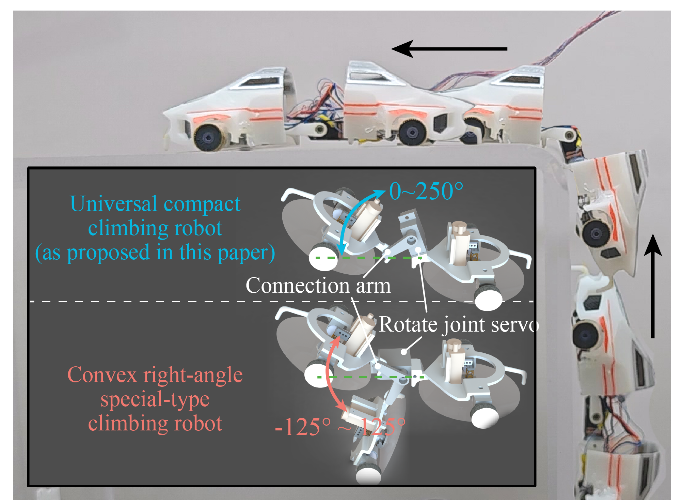


**Fig. S8.** **A schematic diagram of the specialized acoustic adhesion wall-climbing robot traversing a convex right-angle corner.** To minimize robot's size for climbing confined spaces (e.g., aircraft engine interiors), the robot proposed in paper incorporates an angled configuration between its surface-traversing servo and connect ion arm, as shown in the upper of inset. This design achieves an upward articulation range of 250° from the initial posture. Conversely, a parallel arrangement of the servo and arm (lower of inset) enables downward rotation, shifting the overall angular range to -125° to 125°. However, this parallel configuration increases the robot's overall dimensions, significantly compromising its maneuverability within confined inspection environments compared to the integrated angled design.

**Supplementary movie**

**Movie S1.** The process of climbing. The climbing robot went up a step, climbed across non-continuous surfaces, and drove on vertical walls and inverted ceilings without wires, it took university logo at the end. Robot paths and motions were generated with AI assistance, which makes it easy to control the robot to accomplish these complex motions.

**Movie S2.** Experiments about vibrational adhesion pad. The pad’s controllability and maximum force were tested. The adhesion could be controlled by turning the vibration motor on or off. The pad carried a 360 g payload, 180 times its weight. The air layer thickness was measured by high-speed camera and 3D laser scanner.

**Movie S3.** The load ability of the robot. We tested robot’s max load ability on wall and ceiling. It carried a 70 g payload at 70 mm/s on both wall and ceiling.

**Movie S4.** Demonstration of cross-surface climbing robot. Our robot climbed into a 3D maze and moved across the obstacles to reach the endpoint with AI integration. It indicates the great stability, agility, and intelligence of our robot.

**Supplementary materials reference**

[1] S. Ramanarayanan and A. L. Sánchez, "On the enhanced attractive load capacity of resonant flexural squeeze-film levitators," AIP Advances, vol. 12, no. 10, 2022, doi: 10.1063/5.0106730.
